# Am I Winning or Losing? Probing the Appraisal of Partial Wins via Response Vigor

**DOI:** 10.1007/s10899-023-10216-z

**Published:** 2023-06-03

**Authors:** Zhang Chen, Charlotte Eben, Christina B. Reimer, Frederick Verbruggen

**Affiliations:** Department of Experimental Psychology, Ghent University, Henri Dunantlaan 2, 9000 Gent, Belgium

**Keywords:** Appraisal, Response vigour, Partial wins, Losses disguised as wins, Gambling

## Abstract

Attempts to obtain rewards are not always successful. Despite investing much time, effort, or money, sometimes individuals may not obtain any reward. Other times they may obtain some reward, but the obtained reward may be smaller than their initial investment, such as partial wins in gambling. It remains unclear how such ambiguous outcomes are appraised. To address this question, we systematically varied the payoffs for different outcomes in a computerized scratch card task across three experiments. To test outcome appraisal, we used response vigor as a novel proxy. In the scratch card task, participants turned three cards one by one. Depending on the turned cards, they either received an amount that was higher than the wager (win), an amount lower than the wager (partial win), or nothing (loss). Overall, participants responded to partial wins more slowly than losses, but more quickly than wins. Partial wins were therefore appraised to be better than losses, but worse than wins. Importantly, further analyses showed that outcome appraisal was not based on the net win or loss amount. Instead, participants primarily used the configuration of turned cards as a cue for the relative rank of an outcome within a specific game. Outcome appraisals thus utilize simple heuristic rules, rely on salient information (such as outcome-related cues in gambling), and are specific to a local context. Together, these factors may contribute to the misperception of partial wins as real wins in gambling. Future work may examine how outcome appraisal may be modulated by the salience of certain information, and investigate the appraisal process in contexts beyond gambling.

**Supplementary Information:**

The online version contains supplementary material available at 10.1007/s10899-023-10216-z.

## Introduction

Individuals generally seek to obtain rewards (and avoid punishments), but do not always succeed. Such successes and failures in obtaining rewards serve as important teaching signals that enable individuals to learn to adapt their behaviors to a certain environment (Sutton & Barto, 2018). In addition to facilitating learning, these events also have more immediate influences on subsequent motivated behaviors (e.g., Papini, 2003). To understand reward pursuit behavior, it is therefore important to understand how individuals react to successes and failures in obtaining rewards.

Appraisal theories of motivated behavior and emotion propose that how individuals react to outcomes of reward pursuit is largely determined by their appraisals of the outcomes (Scherer & Moors, 2019; Ridderinkhof, 2014; Moors et al., 2021). In the current paper, we thus focus on outcome appraisal in reward pursuit, using gambling as a case study. When gambling, people routinely fail to obtain monetary rewards, as most bets result in losses. Despite the negative expected value of most gambling products (i.e., gamblers on average always lose money), some gamblers persistently engage in gambling (Calado & Griffiths, 2016), which may lead to mounting financial losses and other gambling-related problems (American Psychiatric Association, 2013). Given the ubiquity of gambling and the harms it causes in some individuals, it is important to understand how individuals appraise wins and losses when gambling, as specific instances of successes and failures in obtaining rewards.

In what follows, we first briefly introduce the general theoretical framework of appraisal accounts. We then apply appraisal theories to the gambling context, and argue that outcome appraisal can influence how individuals react to wins and losses in gambling. We then end with the question of the current research, namely how people appraise partial wins, an ambiguous yet common type of outcome in gambling.

### Appraisal Theories of Motivated Behavior and Emotion

Appraisal theories of motivated behavior and emotion assume that the appraisal of an event determines how individuals react to it (Scherer & Moors, 2019; Ridderinkhof, 2014; Moors et al., 2013). When an event is perceived, it is very rapidly appraised, or evaluated, by an individual. The appraisal process gives rise to one or more motives, or action tendencies with certain urgency and strength (Ridderinkhof, 2014; Frijda et al., 2014). Appraisal thus provides motivation for action. Furthermore, event appraisal can lead to changes in other components, such as physiological responses and motor expressions (e.g., facial, vocal, and gestural). The synchronized changes in these components may give rise to a certain subjective feeling or emotion (Scherer & Moors, 2019). As such, event appraisal underlines motivated behavior and emotion triggered by an event.

An event can be appraised based on multiple criteria (Scherer & Moors, 2019). According to multiple appraisal theories, the most central appraisal is whether an event promotes or obstructs one’s goal (e.g., Ridderinkhof, 2017; Ellsworth & Scherer, 2003; Frijda et al., 2014). For instance, the appraisal account by Frijda and colleagues posits that when pursing a certain goal, such as when trying to obtain a reward, individuals constantly monitor the discrepancy between current state and desired state (Frijda, 2010; Frijda et al., 2014). The discrepancy between the two states leads to a state of action readiness, which aims to reduce this discrepancy. When the discrepancy between current state and desired state is large (i.e., one’s goal is obstructed), the strength and urgency of the state of action readiness is high, which can be behaviorally manifest in vigorous responses (e.g., responses executed with more force and a higher speed etc). In contrast, when the discrepancy is small or non-existent (i.e., an event promotes one’s goal), the strength of the state of action readiness is low, and subsequent behaviors may be less vigorous. Other theoretical accounts, such as Carver’s, also emphasize the central role of perceived discrepancy in influencing motivated behavior, although the exact proposed mechanism differs (Carver & Scheier, 2001; Carver, 2003; Carver & Scheier, 1990; Carver, 2004). Under Frijda’s account, response vigor hence provides an unobtrusive, albeit indirect measure of the appraised discrepancy between current and desired state (Frijda, 2010; Frijda et al., 2014).

### Appraising Outcomes in Gambling

When gambling (i.e., a type of reward pursuit activity), the desired state is often to win money in a game. Thus, when a gambler loses, there is a discrepancy between the current state (i.e., losing) and the desired state (i.e., winning). Appraisal theories predict that responses following losses will be more vigorous than those following wins. This prediction has been largely corroborated in gambling situations. In real and simulated gambling, as well as in gambling analogue tasks, people tend to initiate a new round more quickly after a loss than after a win (e.g., Stange et al., 2016, 2017; Chen et al., 2022, 2020; Detez et al., 2019; Ferrari et al., 2022) and after a non-gamble outcome (Verbruggen et al., 2017; Eben et al., 2020).

Frijda’s account (and appraisal theories more generally) further predicts that response vigor crucially depends on an individual’s appraisal of the current and desired state (Frijda et al., 2014). For objectively the same outcomes (i.e., those with the same win or loss amounts), the appraisals of the outcomes may still differ, and response vigor following these outcomes will accordingly also differ. This prediction has been corroborated by a recent study using computerized scratch card games (Chen et al., 2020). This study included different types of losses that were objectively the same - participants lost the same amount of points. However, the appraised discrepancy was different, and modulated the vigor of ensuing responses. More specifically, when participants had a high expectation of winning before eventually losing (due to the order in which symbols appeared; Bossuyt et al., 2014), they responded more quickly compared to when they did not expect to win. This effect occurred presumably because an elevated expectation of winning corresponded to a better desired state, which in turn translated into a larger discrepancy when participants eventually lost. When participants perceived a loss to be proximal to a win (because the loss shared many visual features with the win), they responded more slowly compared to when they perceived a loss to be distal from a win (because the loss shared few visual features with the win; for a similar finding, see Belisle & Dixon, 2016). A loss that is proximal to a win may indicate a subjectively better current state, and the discrepancy between the current and the desired state might therefore be smaller. Thus, objectively the same outcomes (i.e., losing the same amount of points) were appraised differently, depending on the cues that accompanied the losses (Chen et al., 2020).

Some gambling products also include other types of ambiguous outcomes, that may allow for different appraisals. For instance, in some multi-line video slot machines, gamblers can bet on multiple lines within a single game, and can win or lose credits on each line. When they win on some (but not all) of the pay lines, the amount of credits they ’win’ may be smaller than the amount they initially have wagered, so they effectively lose money. However, the machines still present the same celebratory visual and auditory feedback as in actual wins (i.e., when the win amount is larger than the wager amount). In such cases, the losses are said to be ’disguised’ as wins (Dixon et al., 2010). Such partial win outcomes^1^ (i.e., ’winning’ back part of the wager) present an interesting case, because their ambiguous nature may allow for different appraisals. On the one hand, people may incorporate the wager into their appraisals of partial wins, and correctly identify them as losses (although partial wins may still be appraised as better than full losses, and the former often entails smaller net losses than the latter). On the other hand, the appraisal process may be rather ’myopic’, and fail to take the wager amount into account. In the latter case, people may incorrectly perceive partial wins to be actual wins.

Previous work has examined the effects of losses disguised as wins (LDWs), a prominent type of partial wins, in simulated slot machine gambling. Overall, gamblers seemed to largely miscategorize LDWs as actual wins. LDWs tend to trigger similar psychological, behavioral and physiological responses as wins, rather than losses (for a review, see Barton et al., 2017). More directly relevant to the current research, players initiated a new spin more slowly after LDWs than after regular losses in (simulated) slot machines, and the length of the pauses was comparable or even longer after LDWs compared to after actual wins of equivalent sizes (Dixon et al., 2014; Templeton et al., 2015). Together, these results suggested that people incorrectly perceived LDWs as actual wins. However, in these studies, the slot machines presented celebratory audiovisual stimuli after wins and LDWs, whereas after losses, the machines went into a state of silence. Using these realistic feedback events is a strength of these prior studies (Dixon et al., 2014; Templeton et al., 2015), as the celebratory audiovisual feedback paired with LDWs might be important contributors to the miscategorization of LDWs as wins (Dixon et al., 2014, 2015). However, since the feedback events differ after the outcomes, it remains unclear to what extent the observed effects are driven by outcome appraisals, or the audiovisual stimuli instead. The current research aims to address this question, by using minimal and matched feedback after different outcomes. Understanding the appraisal of partial wins will help us better understand their effects on gambling behavior. It will also shed light on the broader question of what information is incorporated into outcome appraisal in gambling.

### The Current Research

We examined how partial wins may be appraised, by using how quickly participants responded (i.e., response vigor) following different outcomes as a novel proxy for outcome appraisal. More vigorous responses indicate a larger discrepancy between current and desired state, and thus a less desirable outcome. This theoretical assumption has been corroborated by previous empirical findings (see above). Using response vigor as a proxy confers several benefits, compared to other measures of appraisal such as self-reports (Bossuyt et al., 2014). Firstly, response vigor can be recorded unobtrusively without interrupting the task. Secondly, appraisal processes are often assumed to proceed quickly without much deliberation (Frijda et al., 2014; Lambie & Marcel, 2002). Response vigor may be better able to capture one’s initial appraisals, compared to self-reports, which may allow for more deliberation and thus revision of one’s initial appraisals. Lastly, in gambling research, games with fast speeds of play (i.e., short intervals between two rounds) are often associated with problem gambling (Harris & Griffiths, 2018). In continuous forms of gambling such as slot machines or instant scratch cards, the speed of play is partly under the control of gamblers themselves, as they can decide themselves on how quickly to start a new round. Using response vigor as the dependent variable will thus provide information on this important aspect of gambling behavior as well.

Participants played a computerized scratch card game (Chen et al., 2020). Although partial wins such as LDWs are often examined in multi-line video slot machines, other gambling products such as scratch cards also contain such ambiguous outcomes (Walker et al., 2022). Examining the effects of partial wins beyond slot machine gambling thus seems worthwhile. In each round of our game, participants spent a certain amount of money to ’buy’ a game (i.e., the wager). They then turned three cards one by one. Based on the turned cards, they either won an amount that was higher than the wager (i.e., wins), lost the whole wager (i.e., losses), or ’won’ an amount that was smaller than the wager (i.e., partial wins). The feedback was kept minimal in each game, with no winning sounds and visuals. By systematically varying the payoffs for different outcomes and recording response vigor (as a proxy for outcome appraisal), we examined how people appraised partial wins.

## Experiment 1

Prior to Experiment 1, we conducted a pilot study with 51 participants on Prolific.co to test the experimental procedure. All materials, raw data and results of this pilot study (and all three experiments in this manuscript) can be found in the OSF repository (https://osf.io/5j6z8/). Overall, the results of the pilot study were descriptively in line with the results observed later on in Experiment 1. Encouraged by the initial findings, we conducted Experiment 1 with a larger sample size (N $\approx$ 100), to enable a direct comparison with a previous experiment that has used the same task but without partial wins (Experiment 1 of Chen et al., 2020).

### Methods

#### Participants

Participants who met the following criteria on Prolific.co were eligible to participate: (1) between 18 and 50 years old; (2) spoke English fluently; (3) did not participate in any previous studies by the first author on Prolific.co; and (4) had at least 70% of previous submissions approved by researchers. In total, 101 participants (51 males, 50 females; $M_{age} = 29.7$, $SD_{age} = 7.9$) took part in the experiment. This sample size (around 100 participants) was based on previous work that used the same task (Chen et al., 2020), to facilitate the direct comparison between the two experiments.

#### Apparatus and Materials

This experiment was programmed in jsPsych (version 6.0.5, de Leeuw, 2015), and ran in Google Chrome or Firefox, as other web browsers may have compatibility issues. Participants could use either a desktop or a laptop to do the experiment.

**Fig. 1:**
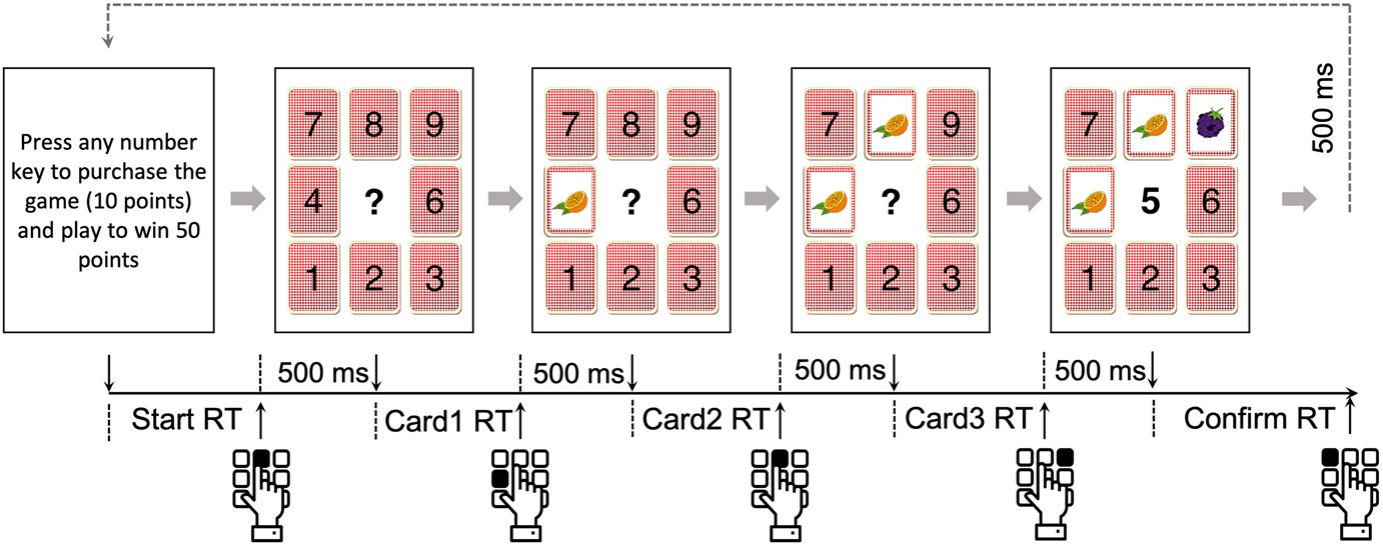
The trial procedure in the scratch card task used in Experiments 1 and 2

In the scratch card task, we used two types of cards (either red or blue, 180 $\times$ 250 pixels) to designate low- and high-amount games (see below). The assignment of card colors to amount levels was randomized across participants. For each game, eight cards (all red or all blue) were presented in a 3 $\times$ 3 grid, with no card presented in the center (see Fig. 1). Cards were separated from each other by 40 pixels horizontally and by 15 pixels vertically. A number (1 till 4, and 6 till 9) was superimposed on the back of each card. When turned, the front of a card contained a drawing of either a strawberry, some grapes, or an orange (picture number 381, 620, and 695 from Duñabeitia et al., 2018). We used these three pictures to create four types of outcomes, namely AAA (i.e., all three cards contained the same drawing), AAB (i.e., the third card differed from the first two), ABB (i.e., the last two cards differed from the first one) and ABC (i.e., all three cards differed from each other). Note that the four types of outcomes denoted general patterns rather than specific cards. For example, the outcome type AAA included instances where all three cards contained a strawberry, all three cards contained grapes, and all three cards contained an orange. Each of the 4 types of outcomes occurred with a 25% probability.

#### Procedure

Participants signed up for the experiment via Prolific.co. They read and agreed to an informed consent before starting the experiment. The rules of the scratch card task were then explained. For AAA, the win amount was five times the wager. For AAB and ABB, the win amount was half of the wager, so participants effectively lost money. For ABC, the win amount was 0. See Table 1 for the wager and presented ’win’ amount for each outcome. To motivate participants, they were told that they could receive an extra bonus based on the accumulated points at the end of the experiment. Every 100 points were worth 50 British pence, and participants could receive a maximum bonus of 3 British pounds.

**Table 1:** Wager and presented ’win’ amounts in the scratch card games in Experiment 1 and 2 and in Experiment 1 of Chen et al. (2020)

	Experiment 1		Experiment 2		Experiment 1 of Chen et al. (2020)	
	Low-Amount	High-Amount	Low-Amount	High-Amount	Low-Amount	High-Amount
Wager	2	10	2	20	2	12
AAA	10 (+ 8)	50 (+ 40)	10 (+ 8)	100 (+ 80)	10 (+ 8)	60 (+ 48)
AAB/ABB	1 (− 1)	5 (− 5)	1 (− 1)	10 (− 10)	0 (− 2)	0 (− 12)
ABC	0 (-2)	0 (− 10)	0 (− 2)	0 (− 20)	0 (− 2)	0 (− 12)

The real net gain/loss amounts are shown in brackets

They then started with the scratch card task (Fig. 1). Each game started with a start screen, with the message “Press any number key to purchase the game (X points) and play to win Y points.” printed on the center of the screen (for low-amount games, X = 2 and Y = 10; for high-amount games, X = 10 and Y = 50; see also Table 1). Participants could press any number key to start the game. 500 ms after starting a game, a 3 $\times$ 3 grid of cards was presented, with a question mark in the center of the grid. Participants could press one of the number keys to turn a corresponding card. The selected card was turned immediately once being selected. The question mark disappeared for 500 ms, during which time participants could not turn the next card yet. We implemented this wait period of 500 ms after each response to ensure that participants had to turn three cards one by one. When the question mark re-appeared, participants could select a second card. When all three cards were turned, the amount of points participants received was shown in the center of the grid. Participants needed to press any number key to confirm the outcome of a game.

The scratch card task contained 240 games in total, with 120 low-amount and 120 high-amount games. For both the low- and the high-amount games, each of the four types of outcomes (i.e., AAA, AAB, ABB and ABC) occurred with a 25% probability. Unbeknownst to the participants, the outcome of each game was pre-determined. For instance, if a game was assigned to the outcome ABC, participants would get three different drawings regardless of which three cards they chose. However, the exact cards turned (i.e., which drawing was assigned the status of A, B or C) was determined randomly for each game.

Participants started with 50 points. To ensure that no participant went bankrupt, they experienced each outcome once from the 2 (amount: high vs. low) by 4 (outcome: AAA, AAB, ABB vs. ABC) combination in the first eight trials. The remaining trials were then randomized such that the balance was never below 0. Throughout the experiment, the total amount of points won so far was presented at the bottom of the screen (not shown in Fig. 1). By the end of the experiment, all participants had 770 points (as the outcomes were predetermined). All of them therefore received a bonus of 3 British pounds, in addition to 3 pounds that they received as compensation for their time.

Participants then filled out the short English version of the UPPS-P impulsive behavior scale (Cyders et al., 2014). This questionnaire was included for another project on individual differences in impulsivity, and the data will not be analyzed here. Participants were debriefed and paid. The current research was approved by the Ethics committee of the Faculty of Psychology and Educational Sciences of Ghent University (No. 2019/86).

### Data Analysis

Data analyses were conducted in R (version 4.0.4, R Core Team, 2021), using packages BayesFactor (version 0.9.12$-$4.2, Morey & Rouder, 2018), ggpubr (version 0.4.0, Kassambara, 2020), hypr (version 0.1.10, Rabe et al., 2020), kableExtra (version 1.2.1, Zhu, 2020), knitr (version 1.2.1, Xie, 2021), lme4 (version 1.1-26, Bates et al., 2020), rmarkdown (version 2.7, Allaire et al., 2021), Rmisc (version 1.5, Hope, 2013), sjPlot (version 2.8.7, Lüdecke, 2021) and tidyverse (version 1.3.0, Wickham, 2019).

#### Data Preparation

Two participants restarted the experiment after finishing 50 and 102 trials, respectively, and were excluded from the analysis. To directly compare the current results with previous work (Chen et al., 2020), we used the same data exclusion criteria. Trials that were not followed by other trials (i.e., the last trial, and a few other trials due to missing data) were first excluded. We then defined one ’episode’ of play as consisting of 8 responses: start a game, turn three cards one by one, confirm the outcome, start the *next* game, and turn the first and second card of the *next* game (see Chen et al., 2020, for a further motivation of this analysis choice). After turning the second card of the *next* game, the outcome of the *next* game is partially revealed. RTs of all eight responses needed to be <= 5 s for an ’episode’ to be included in the analysis. This led to a data exclusion of 5.89%. To achieve reliable estimates, participants needed to have at least 15 ’episodes’ in each cell. One participant did not meet this criterion. The final sample therefore consisted of 98 participants.

#### Data Analysis

Here we focused on the RT of confirming an outcome (hereafter confirm RT) and the RT of starting a new round (hereafter start RT). Both responses immediately followed an outcome, and participants could use any key. The RTs of these two responses therefore served as ’pure’ measures of response vigor. For the RTs of all eight responses in an ’episode’, see the online Supplemental Materials. For each participant, the mean confirm RT and start RT after each outcome was calculated for low-amount and high-amount games separately. Since the results on start RTs were largely in line with those on confirm RTs (unless otherwise noted, see Experiment 3), for simplicity and brevity, we only reported the results on confirm RTs here. For the results on start RTs, see the Supplemental Materials.

To test comparisons of theoretical interest, we used custom contrast coding (Schad et al., 2020), using the R package *hypr*. For the predictor outcome, we created custom contrasts to test two comparisons, namely (1) partial wins (i.e., AAB and ABB^2^) versus wins (i.e., AAA), and (2) partial wins (i.e., AAB and ABB) versus losses (i.e., ABC). For the predictor amount level, we used effect coding (high-amount = 0.5, low-amount = $-$0.5). The two predictors and their interaction were then used to predict the confirm RT (and start RT) in a linear regression. To quantify evidence for null effects, we further computed the Bayes factor for each effect. More specifically, the full linear regression model was compared against a model omitting a specific effect, using the BayesFactor package with the default prior settings (Rouder & Morey, 2012). The resulting Bayes factor (BF) indicates the likelihood of data under a model that contains a specific effect against a model that omits the effect. BF larger than 1 provides support for the alternative hypothesis, whereas BF smaller than 1 provides support for the null hypothesis. To compare specific cells, we used both frequentist and Bayesian t tests (using a Cauchy distribution of width of 0.707 as the prior), and reported Hedges’s gav as the effect size (Lakens, 2013).

### Results

**Fig. 2:**
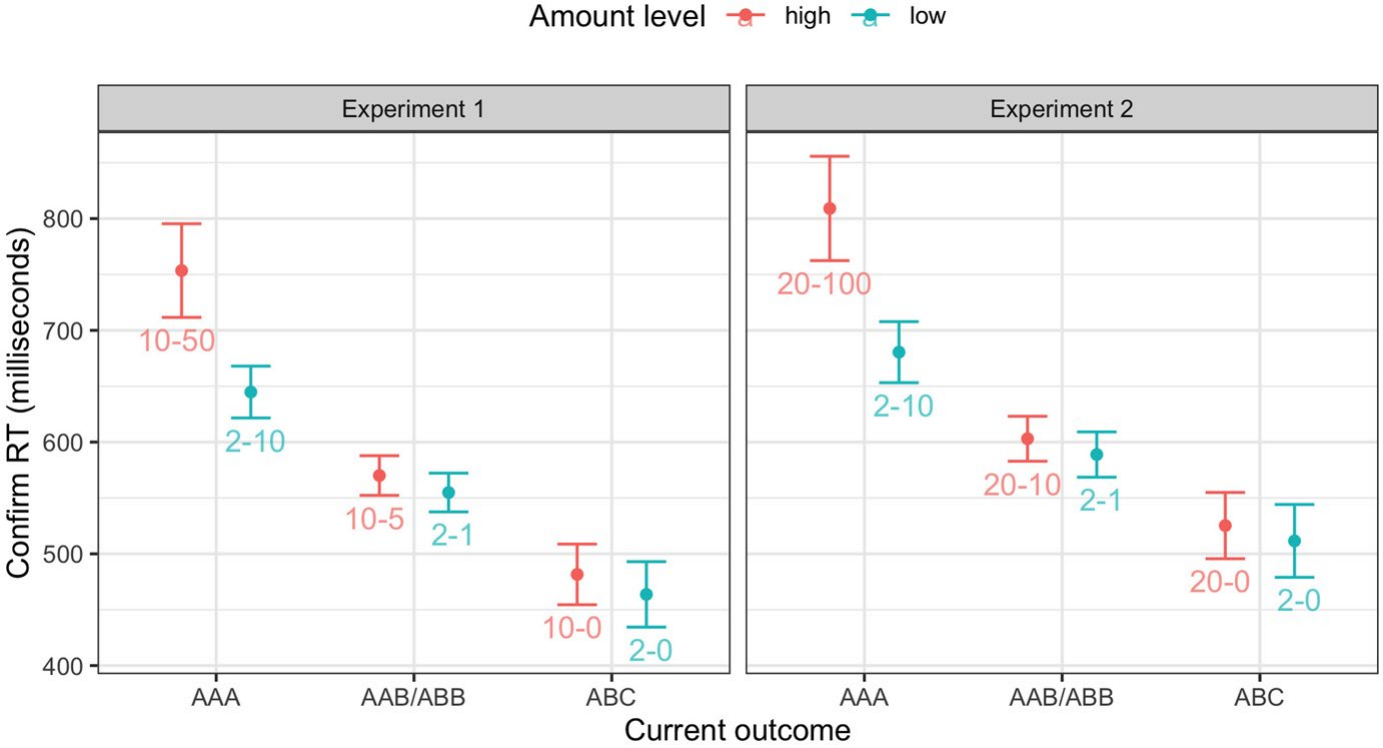
Confirm RTs as a function of game outcome and amount level in Experiment 1 (left) and Experiment 2 (right). Error bars stand for 95% within-subjects confidence intervals. The numbers stand for the wager and the presented ’win’ amount in each game (wager-win)

**Table 2:** Linear regression and pairwise comparisons on confirm RTs in Experiment 1

Linear regression							
Predictor	Estimate	SE	lowerCI	upperCI	t	*p*	BF
Amount	39.25	9.96	19.73	58.77	3.94	< 0.001	201
Partial Win vs. Win	$-$136.68	12.20	$-$160.59	$-$112.77	$-$11.21	< 0.001	$4.80 \times 10^{23}$
Partial Win vs. Loss	89.83	12.20	65.93	113.74	7.37	< 0.001	$1.49 \times 10^{10}$
Amount * (Partial Win vs. Win)	$-$93.51	24.39	$-$141.32	$-$45.69	$-$3.83	< 0.001	143
Amount * (Partial Win vs. Loss)	$-$2.68	24.39	$-$50.50	45.13	$-$0.11	0.912	0.108

Pairwise comparisons							
Comparison	diff	lowerCI	upperCI	t	*p*	BF	gav
Win: High vs. Low	108.7	67.1	150.3	5.19	< 0.001	11979	0.315
Partial Win: High vs. Low	15.2	$-$7.4	37.8	1.33	0.370	0.264	0.055
Loss: High vs. Low	17.9	$-$10.7	46.5	1.24	0.370	0.235	0.082

lowerCI = lower limit of 95% confidence interval; upperCI = upper limit of 95% confidence interval; BF = Bayes factor; gav = Hedges’s average *g*. P values for the pairwise comparisons were corrected for multiple comparisons with the Holm-Bonferroni method

To facilitate the communication of the results, we added the wager and the presented ’win’ amount as subscripts to the card configurations (see also Fig. 2). For instance, $\hbox {AAB}_{10-5}$ means participants wagered 10 points, and ’won’ back 5 points when they got AAB (i.e., a partial win in the high-amount condition).

The results of the linear regression and pairwise comparisons are in Table 2. Participants overall responded more quickly after partial wins (*M* = 562.5, *SD* = 268.1) than after wins (*M* = 699.2, *SD* = 329.4; see Fig. 2). This effect was significantly modulated by the amount level (the ’Amount * (Partial Win vs. Win)’ predictor in Table 2). To break down this interaction effect, we conducted pairwise comparisons, examining the effect of amount level within partial wins and wins separately. While participants responded more slowly after a high-amount win compared to a low-amount win (the ’Win: High vs. Low’ comparison in Table 2), amount level did not modulate confirm RT after partial wins (the ’Partial Win: High vs. Low’ comparison in Table 2).

Participants responded more slowly after partial wins than after losses (*M* = 472.6, *SD* = 205.2), and its interaction with amount level was not statistically significant. The BF provided moderate support for the null hypothesis for the interaction effect. In line with previous findings (Chen et al., 2020; Eben et al., 2020), the loss amount did not modulate confirm RTs for losses (the ’Loss: High vs. Low’ comparison in Table 2), just like it did not modulate confirm RT for partial wins (see the previous paragraph).

The card configurations for partial wins (AAB and ABB) were more proximal to wins (AAA) compared to losses (ABC). Previous work has shown that perceived proximity to win could lead people to slow down (Chen et al., 2020). To isolate the effect of proximity from that of partial wins, we compared the current result against that of Experiment 1 in Chen et al. (2020). In Experiment 1 of Chen et al. (2020), participants wagered 2 or 12 points on each game, and lost the whole wager when they got AAB, ABB or ABC (see Table 1). No partial wins were therefore included. For both experiments, we computed the difference in confirm RTs after AAB/ABB and after ABC. The difference scores were then compared between the two experiments. The difference between AAB/ABB and ABC was substantially larger in the current experiment (*M* = 89.8, *SD* = 127.5) than in Experiment 1 of Chen et al. (2020) (*M* = 35.2, *SD* = 112.3), diff = 54.6, 95% CI = [21.0, 88.2], *t*(192.6) = 3.21, *p* =.002, BF = 18.0, gav = 0.452. The perception that AAB and ABB were proximal to wins alone was thus not sufficient to explain the difference between partial wins (AAB/ABB) and losses (ABC) in the present study. Instead, winning part of the wager back in partial wins additionally reduced response vigor compared to full losses.

On average, the net loss amounts were smaller in partial wins (net losses of 1 and 5 points) than in regular losses (net losses of 2 and 10 points), which might explain why participants responded to partial wins more slowly than regular losses. To explore this possibility, we compared the confirm RT for $\hbox {AAB/ABB}_{10-5}$ (a partial win with a net loss of 5 points) and $\hbox {ABC}_{2-0}$ (a regular loss with a net loss of 2 points). Although the net loss was larger in $\hbox {AAB/ABB}_{10-5}$ than in $\hbox {ABC}_{2-0}$, participants responded to $\hbox {AAB/ABB}_{10-5}$ more *slowly* than $\hbox {ABC}_{2-0}$, diff = 106.4, 95% CI = [72.5, 140.3], *t*(97) = 6.23, *p* <.001, BF = 931873, gav = 0.424. This difference was reliably larger than that observed in Experiment 1 of Chen et al. (2020), when the effect of proximity was controlled for, diff = 81.9, 95% CI = [37.5, 126.3], *t*(192.4) = 3.64, *p* <.001, BF = 68.2, gav = 0.513. These latency analyses therefore suggest that participants appraised partial wins to be better than losses, even when the former entailed a larger net loss.

### Discussion

Participants responded to partial wins more slowly compared to losses, indicating that they appraised partial wins to be better than losses. Two exploratory analyses ruled out two alternative explanations, namely (1) the difference was solely caused by the perception that AAB/ABB were proximal to wins, and (2) the net losses were smaller in partial wins than in losses. When appraising partial wins, participants thus did not seem to compute the net loss amount based on the wager and the presented ’win’ amount. In other words, they showed a potential neglect of the net outcome in their appraisals.

Participants responded to wins more slowly than partial wins. The presented ’win’ amounts were larger for wins (10 and 50 points) than for partial wins (1 and 5 points). Participants might appraise the outcomes purely based on the presented ’win’ amounts, and the larger ’win’ amounts in real wins might therefore make it to be appraised as more favorable than partial wins. To test this possibility, Experiment 2 presented (across some trials) the same ’win’ amount for both partial wins and wins.

## Experiment 2

Experiment 2 was a replication and extension of Experiment 1. We changed the payoffs, so that for the high-amount games, participants needed to pay 20 points. They won 100 points for AAA (wins), and 10 points for AAB and ABB (partial wins), and 0 point for ABC (losses). For the low-amount games, the payoffs remained the same (see Table 1). Since participants received 10 points for the wins in the low-amount condition and the partial wins in the high-amount condition, we could directly compare wins and partial wins when the presented ’win’ amount was matched.

### Methods

#### Participants

The same eligibility criteria as in Experiment 1 were used to recruit participants from Prolific.co. We initially planned to recruit around 100 participants. However, initial inspection of the data during data collection revealed that many participants had a large number of missing trials, possibly due to server issues. To compensate for the reduction in statistical power, we therefore added 20 slots on Prolific.co. In total, 119 participants (before exclusion; 65 males, 54 females; $M_{age} = 29.4$, $SD_{age} = 7.9$) participated.

#### Apparatus, Materials and Procedure

The same apparatus, materials and procedure as in Experiment 1 were used. The only difference was that the payoff for the high-amount games was changed (Table 1).

### Data Analysis

We used the same data preparation and analysis procedure as in Experiment 1. Data from 15 participants were excluded, due to insufficient number of observations, leaving 104 participants in the final analysis.

### Results

**Table 3:** Linear regression and pairwise comparisons on confirm RTs in Experiment 2

Linear regression							
Predictor	Estimate	SE	lowerCI	upperCI	t	*p*	BF
Amount	42.67	10.89	21.32	64.01	3.92	< 0.001	201
Partial Win vs. Win	$-$148.87	13.34	$-$175.01	$-$122.73	$-$11.16	< 0.001	$4.39 \times 10^{23}$
Partial Win vs. Loss	77.48	13.34	51.35	103.62	5.81	< 0.001	$1.25 \times 10^{6}$
Amount * (Partial Win vs. Win)	$-$114.45	26.67	$-$166.72	$-$62.17	$-$4.29	< 0.001	948
Amount * (Partial Win vs. Loss)	0.44	26.67	$-$51.84	52.72	0.02	0.987	0.120

Pairwise comparisons							
Comparison	diff	lowerCI	upperCI	t	*p*	BF	gav
Win: High vs. Low	128.6	83.3	173.9	5.64	< 0.001	80984	0.292
Partial Win: High vs. Low	14.2	$-$4.8	33.1	1.48	0.284	0.312	0.044
Loss: High vs. Low	13.7	$-$15.6	43.1	0.93	0.356	0.165	0.048

lowerCI = lower limit of 95% confidence interval; upperCI = upper limit of 95% confidence interval; BF = Bayes factor; gav = Hedges’s average *g*. P values for the pairwise comparisons were corrected for multiple comparisons with the Holm-Bonferroni method

Experiment 2 largely replicated the results of Experiment 1 (Table 3). Participants responded more quickly after partial wins (*M* = 595.9, *SD* = 315.2) than after wins (*M* = 744.8, *SD* = 427.5), and this effect was moderated by amount level. Pairwise comparisons showed that amount level modulated response vigor after wins, but not after partial wins. Further replicating Experiment 1, participants responded to partial wins more slowly than losses (*M* = 518.4, *SD* = 272.5), and this effect was not modulated by amount level.

Directly comparing the current experiment with Experiment 1 of Chen et al. (2020) similarly revealed that the difference between AAB/ABB and ABC was larger in the current experiment than in Experiment 1 of Chen et al. (2020), diff = 42.2, 95% CI = [4.14, 80.4], *t*(184.2) = 2.19, *p* =.030, BF = 1.39, gav = 0.304. While the p-value was statistically significant, the BF was inconclusive. However, since both the direction and magnitude of the effects were similar in Experiments 1 and 2 (of this study), we can conclude that the difference between partial wins and losses is not entirely due to proximity per se.

As in Experiment 1, we compared $\hbox {AAB/ABB}_{20-10}$ (a partial win with a net loss of 10 points) with $\hbox {ABC}_{2-0}$ (a regular loss with a net loss of 2 points). Participants again responded more slowly after $\hbox {AAB/ABB}_{20-10}$ than after $\hbox {ABC}_{2-0}$, diff = 91.4, 95% CI = [56.0, 126.8], *t*(103) = 5.12, *p* <.001, BF = 9858, gav = 0.311. This difference was again reliably larger than that in Experiment 1 of Chen et al. (2020), diff = 67.0, 95% CI = [21.4, 112.6], *t*(197.4) = 2.90, *p* =.004, BF = 7.31, gav = 0.403. The different net loss amounts thus cannot explain the difference between partial wins and losses.

The new comparison of interest was between $\hbox {AAA}_{2-10}$ and $\hbox {AAB/ABB}_{20-10}$, where the ’presented’ win amount was 10 points in both cases. Participants responded to $\hbox {AAA}_{2-10}$ more slowly ($M = 680.5$, $SD = 376.6$) compared to $\hbox {AAB/ABB}_{20-10}$ ($M = 603.0$, $SD = 318.8$), diff = 77.5, 95% CI = [44.1, 110.9], t(103) = 4.60, $p <.001$, BF = 1321, gav = 0.222. Thus, even when the presented ’win’ amount was matched, participants still responded to regular wins more slowly than partial wins. The presented ’win’ amount alone cannot explain the difference between partial wins and wins. All analyses on start RTs yielded the same pattern of results (see the Supplemental Materials).

### Discussion

Using different payoffs, Experiment 2 largely replicated the results of Experiment 1. Participants responded to partial wins more slowly than losses, which cannot be explained by the proximity of AAB/ABB to wins alone. Proximal losses are regular losses that share many visual features with wins, although objectively they are the same with other regular losses in terms of the net loss amount (Chen et al., 2020). Partial wins in Experiments 1 and 2 cannot be equated with proximal losses, as participants won half of the wager back for partial wins (but not for regular losses). The comparisons against Experiment 1 of Chen et al. (2020) supported this idea that partial wins are not simply proximal losses, as the difference between partial wins and losses remained even after controlling for proximity. Net loss amounts also cannot explain the difference between partial wins and losses, as participants responded to partial wins more slowly than losses even when the former entailed a larger net loss. Lastly, participants responded to partial wins more quickly than wins, even when the presented ’win’ amount was matched. The difference between partial wins and wins was thus not purely driven by the presented ’win’ amount.

We note at least two remaining differences between partial wins and wins. First, participants lost points for partial wins, but won points for real wins. Their appraisals of the outcomes may take the net win or loss into account, but not the exact amount, as previous work has shown that the valence and magnitude of an outcome can be coded independently (Yeung & Sanfey, 2004). Partial wins might therefore be appraised as worse than wins. A second possibility is that in the current setup, wins were always the best outcome, as indicated by the AAA card sequence. In contrast, partial wins were always the second best outcome, as indicated by the AAB/ABB card sequences. Partial wins may therefore be appraised as worse than wins, because participants used the card configurations (rather than the presented ’win’ amounts) as cues to determine how good an outcome was among all possible outcomes. Experiment 3 tested these two possibilities.

## Experiment 3

Experiment 3 aimed to examine the relative contribution of the net win/loss and the card sequences in the appraisal of partial wins, as explained above. We included three types of games, and varied the payoff structure (See Table 4) to test four comparisons of theoretical interest (see Table 5).

**Table 4:** Wager and presented ’win’ amount in the scratch card games in Experiment 3

	Type 1	Type 2	Type 3
Wager	30	10	10
AAA	60 (+ 30)	60 (+ 50)	20 (+ 10)
AAB/ABB	20 (− 10)	20 (+ 10)	0 (− 10)
ABC	0 (− 30)	0 (− 10)	0 (− 10)

The net gain/loss amounts are presented in parentheses

**Table 5:** Four pairwise comparisons and the aim of each comparison in Experiment 3

Comparison	Aim of the comparison
(1) $\hbox {AAB/ABB}_{30-20}$ vs. $\hbox {AAA}_{10-20}$	$\hbox {AAB/ABB}_{30-20}$ is a partial win, while $\hbox {AAA}_{10-20}$ is a real win. Both outcomes have the same presented ’win’ amount, but the card configurations differ. This serves to replicate the same comparison in Experiment 2
(2) $\hbox {AAB/ABB}_{30-20}$ vs. $\hbox {AAB/ABB}_{10-20}$	$\hbox {AAB/ABB}_{30-20}$ is a partial win, while $\hbox {AAB/ABB}_{10-20}$ is a real win. Both outcomes have the same presented ’win’ amount and card configuration, with only the wager amount being different. This is to test whether participants incorporate the net win/loss into their appraisals
(3) $\hbox {AAB/ABB}_{10-20}$ vs. $\hbox {AAA}_{10-20}$	Both are real wins, with the same wager and presented ’win’ amount. Only the card configurations differ. This is to test the role of card configurations in outcome appraisal
(4) $\hbox {AAB/ABB}_{30-20}$ vs. $\hbox {AAB/ABB}_{10-0}$	$\hbox {AAB/ABB}_{30-20}$ is a partial win, while $\hbox {AAB/ABB}_{10-0}$ is a real loss. Both outcomes have the same card configurations and net loss amounts. This is to compare partial wins with real losses

### Methods

#### Participants

We used Bayesian sequential hypothesis testing to determine the sample size (Schönbrodt et al., 2017). The sampling and data analysis plan was registered (see https://osf.io/hf2nj). We started with 100 participants, and increased the sample size in increments of 25 participants. Participants excluded based on pre-registered criteria were replaced to reach the aimed sample size. At each sample size, we conducted four planned comparisons (see the Pre-registered Analysis section below). We decided to stop data collection if all four BFs reached the thresholds (BF > 10 or BF < 1/10), or if we reached the maximum sample size of 250 participants (which was the case here).

The same eligibility criteria as in our previous experiments were used to recruit participants on Prolific.co. In total, 264 participants took part in this experiment. Data from 14 participants were excluded (see the Data Preparation section below). The final sample consisted of 250 participants (132 males, 114 females, 3 non-binary, and 1 did not report their gender; $M_{age} = 27.69$, $SD_{age} = 7.73$).

#### Apparatus, Materials and Procedure

**Fig. 3:**
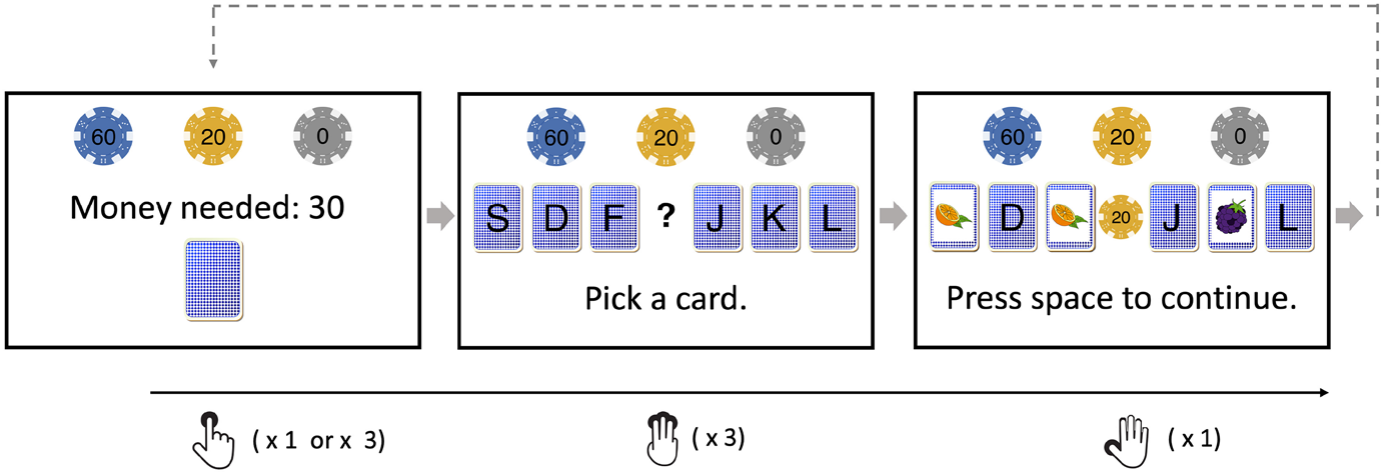
The trial procedure in the scratch card task used in Experiment 3. This example shows an AAB/ABB outcome in a Type 1 game. In Type 2 and Type 3 games, participants needed 10 pence for each game. The three coins (representing potential win amounts) were also adjusted according to the payoff structure in Table 4

The experiment was programmed in jsPysch (version 6.2.0, de Leeuw, 2015). The same materials as in Experiments 1 and 2 were used, but we made several modifications to the task to address some potential limitations of the previous task version (see Fig. 3 for an illustration of the trial procedure).

Participants first received instructions on the rules and the payoff structure of the game. To make the outcomes more distinguishable, we used three types of chips with different colors. Each chip was worth either 60, 20 or 0 British pence (see Fig. 3), with the value printed on each chip. These chips were used to indicate the potential win amounts for different outcomes. Participants were told that the money they had by the end of the experiment would be paid to them as an extra bonus (maximum 1.5 British pounds).

The start screen of each game informed participants which type of game they were going to play, by showing either a blue, green or red card. The assignment of card colors to game types was randomized across participants. The cost of the game was also shown, being either 10 (Type 2 and Type 3 games) or 30 (Type 1 games) pence. Importantly, to start a game, participants needed to press the J key to insert 10 pence each time. For a game that cost 10 pence (Type 2 & 3), they therefore had to press the J key once; for a game that cost 30 pence (Type 1), they had to press the J key three times. We implemented this procedure to make the wagers of the games more salient.

After they had ’inserted’ enough money, the game started. Instead of showing 8 cards in a 3 by 3 grid, we presented 6 cards horizontally and asked participants to use S, D, F and J, K, L to turn the cards. The reason for making this change was because turning cards in the 3 by 3 grid was only intuitive with a number pad. Since not all keyboards have number pads, some participants of previous experiments could have been forced to use the number keys above the letter keys, where the mapping between the key positions and the card positions would be less straightforward. We therefore used S, D, F and J, K, L, keys that are in the same locations in most keyboard layouts. Similar to Experiments 1 and 2, we inserted a 500-ms wait period after each response to ensure that participants turned the cards sequentially.

After participants had turned three cards, a chip was shown in the middle of the cards. Participants needed to press the space bar to continue. In line with Experiments 1 and 2, we focused on the RT of this ’confirm’ response. Throughout a game, the chips participants could win for each outcome were displayed at the top of the screen as a reminder. The total amount of money was displayed at the bottom of the screen (not shown in Fig. 3).

Each of the three types of games occurred 40 times, with the four types of outcomes (i.e., AAA, AAB, ABB and ABC) occurring equally often, for 10 times each. The whole task contained 120 games. After the scratch card task, participants were asked to type in the cost of each game, and the amount of money they could win for each outcome in a memory recall task. They then filled out the UPPS-P questionnaire (Cyders et al., 2014), as part of a larger project. The UPPS-P data will not be analyzed here.

### Data Analysis

#### Data Preparation

Data from participants who restarted the experiment after finishing >= 10 trials, or started the experiment >= 3 times were excluded (N = 1). In each game, participants needed to make five or seven consecutive responses (depending on the cost of a game): starting a game (one or three responses), turning three cards one by one, and confirming the outcome. Trials where any of the five or seven responses were above 5000 milliseconds were excluded^3^. To achieve reliable estimates, participants needed to have at least 5 trials left in each cell in the 3 (game type, Type 1, 2, and 3) by 4 (outcome, AAA, AAB, ABB and ABC) design. Data from 13 additional participants were excluded. Excluded participants were replaced until we reached the aimed sample size at each step.

#### Pre-registered Analysis on Confirm RTs

For each participant, we first calculated the mean confirm RT after each outcome in each type of games. AAB and ABB were combined. We then conducted four Bayesian paired-samples t tests. The BFs from these four comparisons were also used to determine the sample size during sequential testing.

For Comparison (1), we compared $\hbox {AAB/ABB}_{30-20}$ with $\hbox {AAA}_{10-20}$. Both outcomes had the same presented ’win’ amount (i.e., 20 pence), but the card configurations differed. Furthermore, $\hbox {AAB/ABB}_{30-20}$ were partial wins, while $\hbox {AAA}_{10-20}$ were real wins. This comparison served to replicate the same comparison in Experiment 2. We predicted that participants would confirm $\hbox {AAB/ABB}_{30-20}$ more quickly than $\hbox {AAA}_{10-20}$.

As discussed above, Comparison (1) confounded two factors (the net win/loss, and the card configurations). Comparisons (2) and (3) served to disentangle these two factors. In Comparison (2), we compared $\hbox {AAB/ABB}_{30-20}$ and $\hbox {AAB/ABB}_{10-20}$. $\hbox {AAB/ABB}_{30-20}$ were partial wins, while $\hbox {AAB/ABB}_{10-20}$ were actual wins (although the win amount was lower than for $\hbox {AAA}_{10-60}$ outcomes). Thus, both outcomes had the same presented ’win’ amount (i.e., 20 pence), and both were the second best outcomes in their respective games. They differed though in the net win/loss, as the cost of the game was different. Since we made the costs of the games salient, we expected participants to incorporate the net win/loss into their appraisals of the outcomes (the first possibility in Experiment 2). Thus, we predicted that participants would confirm $\hbox {AAB/ABB}_{30-20}$ more quickly than $\hbox {AAB/ABB}_{10-20}$.

In Comparison (3), we compared $\hbox {AAB/ABB}_{10-20}$ with $\hbox {AAA}_{10-20}$. Both were real wins, with the same wager and win amount. However, $\hbox {AAB/ABB}_{10-20}$ was the second best outcome in Type 2 games, while $\hbox {AAA}_{10-20}$ was the best outcome in Type 3 games. This allowed us to test whether the relative ’goodness’ of an outcome, as indicated by the card configurations, could influence response vigor, the second possibility posited in Experiment 2. For this comparison, we did not have a specific prediction.

Lastly, in Comparison (4), we compared $\hbox {AAB/ABB}_{30-20}$ and $\hbox {AAB/ABB}_{10-0}$. This served to compare partial wins with regular losses, with both the net loss amount (i.e., losing 10 pence) and the card configurations matched. For this comparison, we expected participants to confirm $\hbox {AAB/ABB}_{30-20}$ more slowly than $\hbox {AAB/ABB}_{10-0}$, as the former were partial wins while the latter were regular losses.

#### Exploratory Analysis on Start RTs

We concluded exploratory (i.e., not preregistered) analyses on the RTs of starting a new round, using the same data preparation and analysis methods as for confirm RTs. Participants needed to press the J key either once or three times to start a game, depending on how much the game cost. We used the RT of the first press on the J key as the dependent variable.

### Results

**Table 6:** Pairwise comparisons on confirm RTs and start RTs in Experiment 3

Pre-registered analyses on confirm RTs							
Comparisons	diff	lowerCI	upperCI	t	*p*	BF	gav
(1) $\hbox {AAB/ABB}_{30-20}$ vs. $\hbox {AAA}_{10-20}$	$-$38.7	$-$69.8	$-$7.7	$-$2.46	0.045	1.35	0.095
(2) $\hbox {AAB/ABB}_{30-20}$ vs. $\hbox {AAB/ABB}_{10-20}$	20.7	$-$3.9	45.4	1.66	0.099	0.272	0.055
(3) $\hbox {AAB/ABB}_{10-20}$ vs. $\hbox {AAA}_{10-20}$	$-$59.5	$-$88.4	$-$30.5	$-$4.05	< 0.001	182	0.149
(4) $\hbox {AAB/ABB}_{30-20}$ vs. $\hbox {AAB/ABB}_{10-0}$	25.5	2.7	48.3	2.21	0.056	0.767	0.068

Exploratory analyses on start RTs							
Comparisons	diff	lowerCI	upperCI	t	*p*	BF	gav
(1) $\hbox {AAB/ABB}_{30-20}$ vs. $\hbox {AAA}_{10-20}$	$-$92.5	$-$119.2	$-$65.8	$-$6.83	< 0.001	$1.03 \times 10^8$	0.231
(2) $\hbox {AAB/ABB}_{30-20}$ vs. $\hbox {AAB/ABB}_{10-20}$	$-$50.0	$-$72.9	$-$27.0	$-$4.29	< 0.001	466	0.127
(3) $\hbox {AAB/ABB}_{10-20}$ vs. $\hbox {AAA}_{10-20}$	$-$42.5	$-$68.1	$-$17.0	$-$3.28	.002	13.1	0.103
(4) $\hbox {AAB/ABB}_{30-20}$ vs. $\hbox {AAB/ABB}_{10-0}$	$-$7.8	$-$29.8	14.2	$-$0.70	0.486	0.092	0.020

lowerCI = lower limit of 95% confidence interval; upperCI = upper limit of 95% confidence interval; BF = Bayes factor; gav = Hedges’s average *g*. P values were corrected for multiple comparisons with the Holm-Bonferroni method, for the pre-registered and exploratory analyses separately

**Fig. 4:**
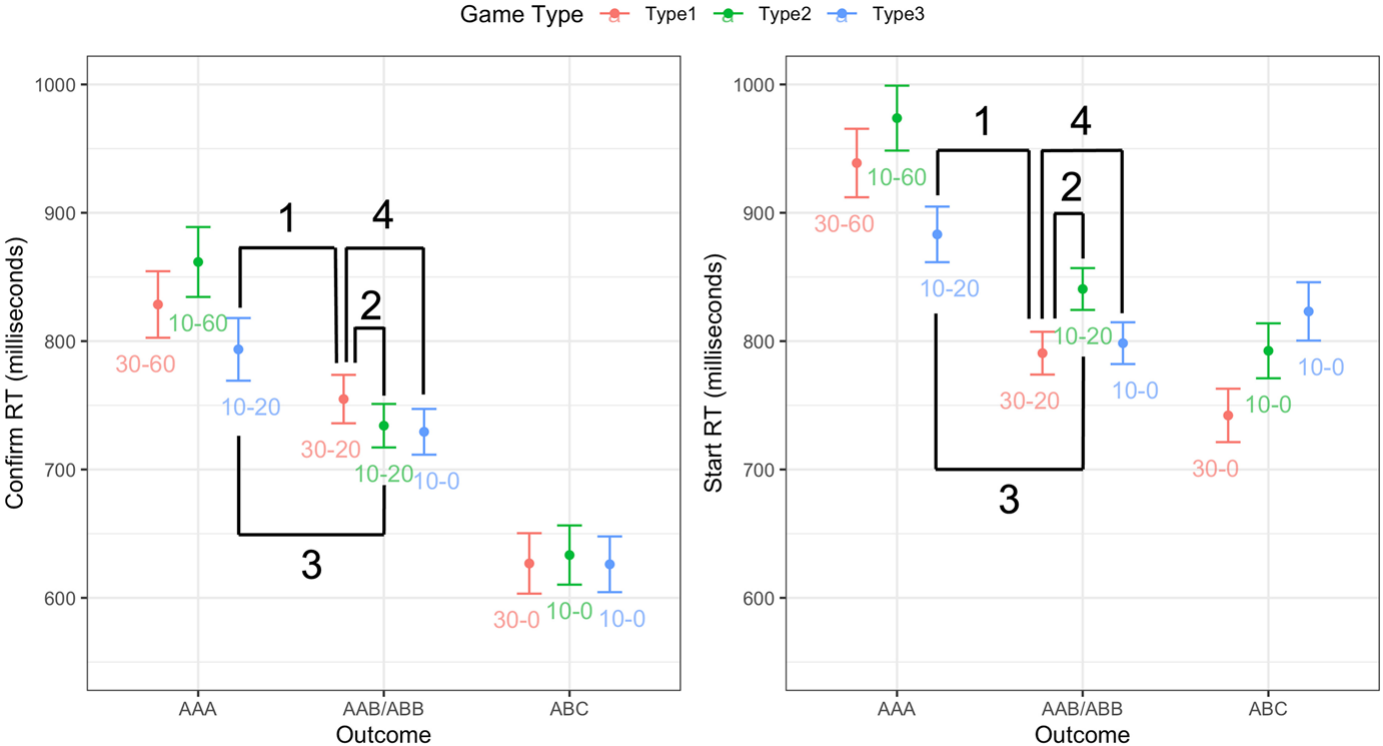
Confirm RTs (left) and Start RTs (right) as a function of outcome and game type in Experiment 3. Error bars stand for 95% within-subjects confidence intervals. The numbers in red, green and blue stand for the wager and the presented ’win’ amount in each game (wager-win). The numbers 1–4 in black show the two cells involved in each of the four comparisons (see Table 6 for the corresponding results) (Color figure online)

Results of the pairwise comparisons are in Table 6. Comparison (1) aimed to replicate the difference between partial wins and wins in Experiment 2. In line with the prediction and replicating the result of Experiment 2, participants confirmed $\hbox {AAB/ABB}_{30-20}$ (*M* = 754.9, *SD* = 386.7) more quickly than $\hbox {AAA}_{10-20}$ (*M* = 793.6, *SD* = 424.3; see also Fig. 4). However, this effect was rather small. Although the difference was statistically significant (after correcting for multiple comparisons), the BF was inconclusive.

Comparison (2) tested whether the difference between partial wins and wins could be explained by the net win/loss in these two types of outcomes. Contrary to our prediction, the comparison between $\hbox {AAB/ABB}_{30-20}$ and $\hbox {AAB/ABB}_{10-20}$ (*M* = 734.1, *SD* = 372.8) revealed moderate support for the null hypothesis. Furthermore, descriptively the effect was in the opposite direction than our initial prediction.

Comparison (3) aimed to test whether the relative standing of an outcome within a game, as indicated by the card configuration, could explain the difference between partial wins and wins. Participants confirmed $\hbox {AAB/ABB}_{10-20}$ more quickly than $\hbox {AAA}_{10-20}$ (*M* = 793.6, *SD* = 424.3). These two outcomes had the same wager amount and the same net win, with the only difference being the card configurations. Card configurations thus influenced the appraisals of outcomes, presumably because they served as salient cues for relatively how good an outcome was within a game.

Comparison (4) compared partial wins and losses, with both the card configurations and the net loss amounts matched. This comparison between $\hbox {AAB/ABB}_{30-20}$ (partial wins) and $\hbox {AAB/ABB}_{10-0}$ (losses) revealed a surprisingly small effect. Participants responded to $\hbox {AAB/ABB}_{30-20}$ more slowly than $\hbox {AAB/ABB}_{10-0}$ (*M* = 729.4, *SD* = 363.8). Although the direction of the effect was in line with Experiments 1 and 2, the p-value was not statistically significant after correcting for multiple comparisons, and the BF was inconclusive.

At the end of the experiment, participants were asked to report the payoffs for each game in a memory task. Inspection of the answers showed that participants made the most errors for Type 3 games, the game type that had different win amounts than the other two. Exploratory analyses using only the participants who remembered the payoffs correctly showed a statistically reliable effect (i.e., p <.05 and BF > 3) for Comparison (4). Comparison (3) was also still statistically reliable in the exploratory analyses (see the Supplemental Materials).

Finally, the exploratory analyses on start RTs showed a somewhat different pattern of results (Table 6 and Fig. 4). Comparison (3) was still statistically reliable, as participants started a new trial more quickly after $\hbox {AAB/ABB}_{10-20}$ than $\hbox {AAA}_{10-20}$. However, for Comparison (2) they now responded more quickly after $\hbox {AAB/ABB}_{30-20}$ than after $\hbox {AAB/ABB}_{10-20}$, suggesting that there was an effect of the overall win/loss. For Comparison (1), the difference between $\hbox {AAB/ABB}_{30-20}$ and $\hbox {AAA}_{10-20}$ was highly reliable. Lastly, for Comparison (4), there was strong evidence for the null hypothesis (the result was the same when only including those who remembered the payoff correctly).

### Discussion

In Experiment 2, participants responded to partial wins more quickly than wins, even though the presented ’win’ amount was matched. Two possible explanations (not mutually exclusive) existed. Participants could use the net win/loss, and/or the card configurations to appraise the outcomes. Experiment 3 aimed to disentangle these two possibilities. The results largely supported the second possibility. For the pre-registered comparisons on confirm RTs, participants responded to $\hbox {AAB/ABB}_{10-20}$ more quickly than $\hbox {AAA}_{10-20}$, even though the two outcomes were matched both in the wager amount and the presented ’win’ amount, with only the card configurations being different. This effect was also found in the exploratory analyses on start RTs. The evidence for the first possibility, namely people took the net win/loss into account in outcome appraisal, was more limited. For confirm RTs, participants did not respond to $\hbox {AAB/ABB}_{30-20}$ more quickly than $\hbox {AAB/ABB}_{10-20}$, suggesting that they initially failed to incorporate the wager into the appraisal, despite the fact that the different wager amounts were made more salient. However, exploratory analyses on start RTs showed that participants started a new trial more quickly after $\hbox {AAB/ABB}_{30-20}$ than after $\hbox {AAB/ABB}_{10-20}$, suggesting the wager might only be incorporated into appraisal when participants started a new round.

The effects in Experiment 3 were relatively small, which may be due to two reasons. First, Experiment 3 included three types of games rather than two, which made the game rules more complex. Indeed, participants tended to confuse the payoff of Type 3 games, which seemed to reduce the difference between partial wins and losses (Comparison (4)) in the full sample. Second, the largest win amounts differed by a ratio of 3:1 (i.e., 60 vs. 20 pence) among different game types in Experiment 3, while they differed by ratios of 5:1 and 10:1 in Experiments 1 and 2. The smaller differences among the game types might have reduced the effect sizes. While bearing these caveats in mind, the overall pattern of the results, together with those from Experiments 1 and 2, supported the proposition that when appraising outcomes, participants initially did not consider the wager, but instead relied on the card configurations as cues for how good an outcome relatively was. We discuss the implications of this finding next.

## General Discussion

’Winning’ part of one’s wager back constitutes an ambiguous outcome that may not be straightforwardly categorized as either a win or a loss. To examine how people appraise such outcomes, we varied the payoffs for different outcomes in a scratch card task across three experiments. We used the latencies of responses following different outcomes as an indirect measure of outcome appraisal. Participants responded to partial wins more slowly than losses, even when partial wins entailed a larger net loss than losses (Experiments 1 and 2). This difference between partial wins and losses remained when we controlled for the effect of proximity either between experiments (Experiments 1 and 2) or within an experiment (Experiment 3). Note though the difference between partial wins and losses in Experiment 3 was small in the whole sample, and was only statistically reliable (post-hoc) in a subset of participants who remembered the payoffs correctly. Participants responded to partial wins more quickly than wins (Experiments 1 and 2), even when the presented ’win’ amounts were matched (Experiment 2). Moreover, this effect was mainly driven by the card configurations (Experiment 3). Overall, this whole pattern of results suggests that participants appraised the outcomes largely based on the card configurations as cues for relatively how good an outcome was in a game, rather than the net win/loss amounts.

When appraising outcomes, participants thus disregarded the wager amount, at least initially. They responded to partial wins more slowly than losses, even when the former entailed a larger net loss (Experiments 1 and 2). Furthermore, in Experiment 3, when both the presented ’win’ amount and the turned cards were controlled for (i.e., $\hbox {AAB/ABB}_{30-20}$ vs. $\hbox {AAB/ABB}_{10-20}$), no effect of the wager amount on confirm RTs was observed. Furthermore, the net loss amount also did not influence confirm RTs after partial wins (AAB/ABB) in Experiments 1 and 2. Thus, the net loss amount had very limited influences on the appraisals of partial wins.

Instead, the whole pattern of results can be most parsimoniously explained by that people mainly appraised outcomes based on their relative ranks in a game. In both Experiments 1 and 2 (and Type 1 and 2 games in Experiment 3), AAA was always the best outcome within a certain game type, AAB/ABB the second best, and ABC the worst. Participants may have used the card configuration as a cue for the rank of a certain outcome among all possible outcomes within a game, and use this information to appraise the outcomes accordingly. This (partly) explains why participants overall responded to partial wins (AAB/ABB) more quickly than wins (AAA), but more slowly than losses (ABC) in Experiments 1 and 2, as the outcomes differed in their ranks within a game. It also explains the results when we compared outcomes between different game types. For instance, in Experiments 1 and 2, participants responded to AAB/ABB more slowly than ABC, even when the former entailed a larger net loss. Since AAB/ABB was ranked the second within its own game, while ABC was ranked the last within its own game, AAB/ABB might therefore be appraised as better than ABC. Similarly, in Experiment 3, participants responded to $\hbox {AAA}_{10-20}$ more slowly than $\hbox {AAB/ABB}_{10-20}$, as the former was ranked the first and the latter the second in their respective games.

Two features of this proposed mechanism are worth noting. First, participants use the rank of an outcome within a specific game, rather than among all games, to appraise how good an outcome is. That is, the context within which an outcome is appraised seems to be local (i.e., within a specific game) rather than global (i.e., within all game types in an experiment). This proposition is broadly in line with some previous work showing that value encoding is highly dependent on its local context (Palminteri & Lebreton, 2021; Madan et al., 2021; Louie & Glimcher, 2012; Rangel & Clithero, 2012). Appraising an outcome in its local context may be beneficial, as it simplifies the appraisal process. Participants can apply the same rule (e.g., AAA = the best, AAB/ABB = the second, ABC = the worst) across different game types. It additionally explains why participants experienced the most difficulties in remembering the payoffs for Type 3 game in Experiment 3, as it was the only game type for which this rule was not applicable. Note that although an outcome was primarily appraised in its local context (i.e., a specific game), comparisons across game types might still be involved. In all three experiments, we observed that confirm RT was modulated by the presented ’win’ amount only after AAA, but not after the other outcomes. This indicates that participants may retain the ranges of possible outcomes across game types, and then appraise an outcome within a specific range based on the game it belongs to (Rangel & Clithero, 2012).

Second, participants mainly used the card configurations to appraise an outcome, rather than other arguably more relevant information, such as the wager and the presented ’win’ amount. In the current setup, participants confirmed an outcome when the three turned cards were presented together with the ’win’ amount. The turned cards were arguably the most salient element of the visual display. The initial appraisal of an outcome (as captured by confirm RTs) thus seemed rather ’myopic’, and only incorporated information that was immediately available at the moment of appraisal. Frijda proposed that appraisals of events often proceeds very quickly without much deliberation or planning (’automatically’, Frijda, 2010; Frijda et al., 2014). We contend that one signature of such ’automaticity’ may be that the appraisal process has a limited temporal scope, and only considers information that is currently available.

The information that people incorporate into the appraisal of an outcome may depend on the task setup. In the current research, we assessed the RTs of two consecutive responses, that of confirming an outcome and starting a new trial, as proxies for the appraisals of the said outcome. In Experiments 1 and 2, results on confirm RTs and start RTs were highly consistent (see the Supplemental Materials). In contrast, in Experiment 3 the results on the two RTs showed a slightly different pattern. Most notably, start RTs showed an influence of the wager amount, while this effect was absent for confirm RTs. One difference between Experiments 1 & 2 and Experiment 3 was that the wager amount was made more salient in Experiment 3, by requiring participants to press the J key either once or three times. It could be that when participants needed to start a new game, the different wager amounts became more salient, thus influenced start RTs but not confirm RTs.

This finding may raise the question of to what extent the current findings are generalizable to partial wins in real gambling products, such as LDWs in multiline video slot machines. After all, here participants received credits to play, and the games overall offered positive expected values (i.e., they won money). In contrast, in real gambling context, gamblers use their own money and tend to lose money in the long run. Furthermore, the net win/loss may be more important in real gambling, as it directly determines the play time. All these factors combined may make the wager more salient in real gambling, thereby allowing gamblers to consider the wager when appraising a particular outcome. However, note that in the current research we largely minimized the visual and auditory feedback associated with winning and losing, yet still observed a reliable effect of the card configuration on outcome appraisal. Real gambling products make more extensive use of such outcome-related cues, and notably pair celebratory audiovisual feedback with both wins and LDWs (Dixon et al., 2014; Templeton et al., 2015). These outcome-related cues may dominate the appraisals of outcomes in real gambling products, to the extent that gamblers disregard their monetary loss (e.g., Dixon et al., 2014, 2015). To reduce such appraisals, regulators may require operators to reduce the use of these cues in gambling products (Dixon et al., 2014, 2015). Furthermore, the wager may be made more salient, for instance by prominently displaying the amount of money bet through the whole round. These measures may facilitate the incorporation of wager amount into outcome appraisal, as the current results suggest.

### Limitations and Future Directions

One important limitation of the current research is that we only used response vigor after an outcome as a proxy for outcome appraisal. Although using response vigor confers multiple benefits (as discussed in the Introduction), it potentially suffers from the problem of ’reverse inference’ (i.e., appraisals influence response vigor, but changes in response vigor may not necessarily reflect changes in appraisals; for a similar issue in inferences based on, e.g., neuroimaging data, see Poldrack, 2006). However, using behavioral measures to capture outcome appraisal seems inevitable, since appraisal is an unobservable mental process. Appraisal theories of emotion posit that appraisal underlies changes in other components, such as physiological responses (Israel and Schönbrodt 2021) and motor expressions such as facial expressions (Gentsch et al., 2015). Future work may use these other measures to capture outcome appraisal (e.g., Wu et al., 2016), and see whether convergent evidence can be obtained.

Second, we did not include other measures that may be of interest in the gambling context. Most notably, the inclusion of partial wins in a gambling product has been shown to lead to an overestimation of the overall winning rate (Jensen et al., 2013; Templeton et al., 2015), which may be one reason why gambling products with partial wins are preferred by gamblers (Dixon et al., 2014). Future work may measure both response vigor following different outcomes and the estimation of winning rate (and other aspects of betting behavior, such as persistence and stake size), and examine whether the appraisal pattern captured by response vigor is related to the magnitude of the overestimation bias and the betting behavior.

**Fig. 5:**
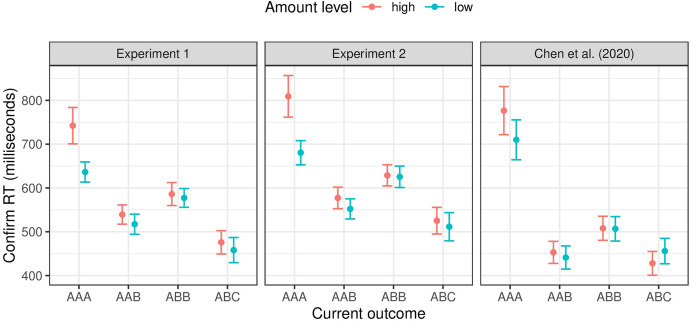
Confirm RTs as a function of game outcome and amount level in Experiment 1 (left), Experiment 2 (middle) and Experiment 1 of Chen et al. (2020). Error bars stand for 95% within-subjects confidence intervals

**Fig. 6:**
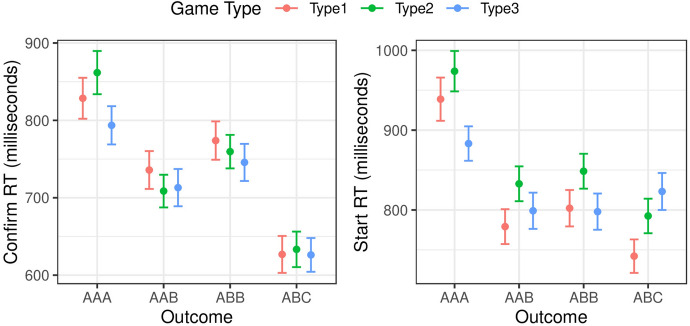
Confirm RTs (left) and start RTs (right) as a function of game type and outcome in Experiment 3. Error bars stand for 95% within-subjects confidence intervals

In this project, we combined AAB and ABB, as we were mainly interested in partial wins. However, the appraisals of AAB and ABB were not entirely the same, as participants confirmed AAB more quickly than ABB (see Fig. 5 and 6 in the Appendix). This replicated the findings from Chen et al. (2020). The perceived discrepancy between current state and desired state may be larger in AAB than in ABB, because participants expected to win before eventually losing in AAB, but not in ABB. This observation suggests that outcome appraisals also incorporate expectancy information (and proximity, see Chen et al., 2020), in addition to the process discussed above. Another caveat by combining AAB and ABB is that partial wins became more frequent than both wins and losses. This may have contributed to the difference between partial wins and wins, as participants might have slowed down after encountering relatively infrequent wins (e.g., surprise). In real gambling, the outcomes may be more unevenly distributed, which may influence response vigor. Furthermore, response vigor in real gambling may be further modulated by outcome streaks (Ferrari et al., 2022) and how much experience one has with gambling (e.g., Chen et al., 2022). These questions can be examined in future research.

Coming back to the question posed in the opening paragraph, namely how people react to successes and failures in reward pursuit, the current research shows that people appraise outcomes of reward pursuit mainly based on outcome-related cues in the gambling context. One remaining question is whether similar processes may be involved in reward pursuit activities other than gambling. For instance, for a student studying for an exam, the amount of effort invested may be conceptualized as a cost (akin to the wager in gambling). The grade obtained is the outcome of reward pursuit, which may be evaluated in a particular context, for instance in comparison to all grades from a certain semester or course. However, in this case, the amount of effort is probably rather salient, and may be incorporated into outcome appraisal (see e.g. Yu et al., 2014, for an effect of expended effort on frustration when reward pursuit attempts were thwarted). One avenue for future work is therefore to examine how individuals may appraise outcomes of reward pursuit in these other contexts.

### Conclusion

During reward pursuit, the acquired outcome may sometimes be smaller than one’s initial investment, but still be better than not getting anything. Using response vigor as a proxy, the current research examined how such ambiguous outcomes are appraised. Participants responded to partial wins more slowly than losses, but more quickly than wins. These differences were mainly driven by the card configurations, rather than the net gain or loss amount. Thus, when appraising an outcome, people disregarded the wager, and primarily used outcome-related cues (i.e., the card configurations) for the relative rank of an outcome within a specific context. The appraisal process appears to follow simple heuristic rules, primarily use information that is immediately available, and is specific to a local context. Future work may further examine how outcome appraisal may be influenced by manipulating the salience of certain information, how it relates to other aspects of reward pursuit behavior, and outcome appraisal beyond the gambling context.

### Supplementary Information

Below is the link to the electronic supplementary material.

Supplementary file1 (PDF 1489 KB)

## Data Availability

All experimental materials, raw data and analysis code can be found at https://osf.io/5j6z8/.

## Appendix

See Figs. 5, 6.

## Footnotes

1 We use the term ’partial win’ to denote outcomes where people ’win’ back part of their initial wager, and the term ’losses disguised as wins’ to specifically denote how such partial win outcomes are implemented in multi-line video slot machines. As mentioned in the main text, losses disguised as wins in slot machines are often accompanied by celebratory visual and auditory feedback, which themselves may influence the appraisal process and subsequent gambling behavior (e.g., Winstanley & Hynes, 2021). Since we are mainly interested in the effect of ’winning’ back part of one’s wager, we refer to such outcomes as ’partial wins’.

2 In the current research, we were mainly interested in the effect of partial wins. The outcome types AAB and ABB were thus collapsed. AAB and ABB were not exactly the same though. In previous work, participants responded more quickly after AAB than after ABB (Chen et al., 2020), which was also observed in all experiments here. See the Appendix for figures where AAB and ABB were presented separately.

3 Note that in Experiments 1 and 2, we defined one ’episode’ of play as from starting the current game till turning the second card in the *next* game, for the sake of consistency with the previous work (Chen et al., 2020). However, since Experiment 3 did not involve between-experiment comparisons, we used a trial as the unit of analysis to retain more data.
