# Supplementary material for: Am I Winning or Losing? Probing the Appraisal of Partial Wins via Response Vigor
Source: J Gambl Stud. 2023 Jun 3;40(1):131–57. doi: 10.1007/s10899-023-10216-z (PMC10904435; doi:10.1007/s10899-023-10216-z)
Supplement: Supplementary file 1 — Supplementary file1 (PDF 1489 KB) [file 10899_2023_10216_MOESM1_ESM.pdf]

Supplemental Materials for *Am I winning or losing? Probing the appraisal of partial wins via response vigor*

Author

Affiliation

Supplemental Materials for *Am I winning or losing? Probing the appraisal of partial wins via response vigor*

Experiment 1

Testing log

Data collection for Experiment 1 took place on 5 Sep 2019. 101 participants signed up and finished the experiment. 2 participants initially signed up for the experiment but did no finish the experiment in time (e.g., timed out). Another 9 participants initially signed up but later returned. No data were registered for the latter 11 participants.

RTs across all stages

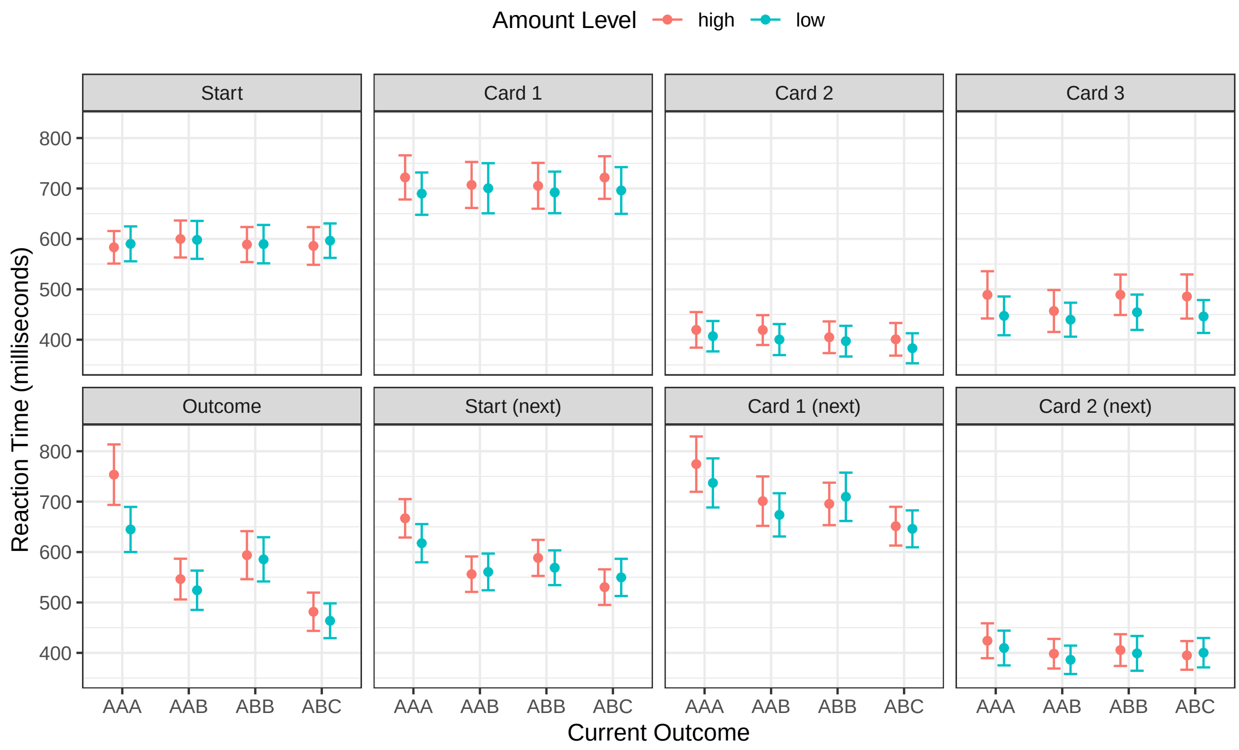

Figure S1

Reaction times of all eight responses within an 'episode' in Experiment 1. Error bars stand for 95% within-subjects confidence intervals.

Figure S1 shows the mean reaction times of all eight responses within one ’episode’ of play, as a function of both the amount level and outcome of the current round.

Analyses on the start RTs

Table S1

Linear regression and pairwise comparisons on start RTs in Experiment 1.

Linear regression							
**Predictor**	**Estimate**	**SE**	**lowerCI**	**upperCI**	**t**	**p**	**BF**
Amount	11.27	6.69	-1.85	24.39	1.69	0.092	0.529
Partial Win vs. Win	-73.74	8.19	-89.82	-57.67	-9.01	< 0.001	2.53×10^{15}
Partial Win vs. Loss	28.49	8.19	12.42	44.56	3.48	0.001	49.9
Amount * (Partial Win vs. Win)	-41.87	16.38	-74.01	-9.72	-2.56	0.011	3.33
Amount * (Partial Win vs. Loss)	26.8	16.38	-5.32	58.97	1.64	0.102	0.508
Pairwise comparisons							
**Comparison**	**diff**	**lowerCI**	**upperCI**	**t**	**p**	**BF**	**gav**
Win: High vs. Low	49.4	17.9	80.9	3.11	0.006	9.979	0.174
Partial Win: High vs. Low	7.5	-7.7	22.7	0.98	0.329	0.178	0.033
Loss: High vs. Low	-19.3	-43.3	4.7	-1.60	0.226	0.381	0.087

Note. lowerCI = lower limit of 95% confidence interval; upperCI = upper limit of 95% confidence interval; BF = Bayes factor; gav = Hedges’s average *g*. P values for the pairwise comparisons were corrected for multiple comparisons with the Holm-Bonferroni method.

The analyses on the start RTs (the "Start (next)" phase in Figure S1) showed consistent results as those observed on confirm RTs, although the effect sizes were overall smaller (see Table S1). To control for the effect of proximity, we similarly computed the difference between AAA/ABB and ABC for both the current experiment and Experiment 1 of Chen et al., 2020. In line with the finding reported in the main text, the difference between AAB/ABB and ABC in start RTs was larger in the current experiment ($M = 28.5$, $SD = 70.3$) than in Experiment 1 of Chen et al., 2020 ($M = -15.3$, $SD = 78.4$), diff = 43.7, 95% CI = [23.0, 64.5], t(197.1) = 4.16, $p < .001$, BF = 381, gav = 0.586. To explore

the effect of net loss amounts, the start RTs for AAB/$ABB_{10\text{-}5}$ and $ABC_{2\text{-}0}$ were compared. Participants descriptively started a new round more slowly after AAB/$ABB_{10\text{-}5}$ than after $ABC_{2\text{-}0}$, diff = 22.6, 95% CI = [1.5, 43.7], t(97) = 2.13, p = .036, BF = 0.958, gav = 0.097, although the Bayes factor was inconclusive and the effect size was rather small. However, this difference was statistically larger than the one observed in Experiment 1 of Chen et al., 2020, diff = 39.5, 95% CI = [8.0, 70.9], t(196.4) = 2.48, p = .014, BF = 2.60, gav = 0.349. The results of both exploratory analyses were therefore replicated on start RTs.

Experiment 2

Testing log

Data collection for Experiment 2 took place on 4 Feb 2020. 117 participants signed up and finished the experiment. 3 participants were timed out, but two of them finished the experiment (resulting in 119 data sets in total). 13 participants initially signed up but later returned. No data were registered for these participants.

RTs across all stages

Figure S2 shows the mean reaction times of all eight responses within one 'episode' of play, as a function of both the amount level and outcome of the current round.

Analyses on the start RTs

The results on start RTs were again consistent with those on confirm RTs (Table S2). The difference between AAB/ABB and ABC in start RTs was again larger in the current experiment (M = 39.1, SD = 91.9) than in Experiment 1 of Chen et al., 2020 (M = −15.3, SD = 78.4), diff = 54.4, 95% CI = [30.9, 77.8], t(200.2) = 4.57, p < .001, BF = 1871, gav = 0.635. For the exploratory analyses on net loss amounts, participants responded to AAB/$ABB_{20\text{-}10}$ more slowly than to $ABC_{2\text{-}0}$, diff = 48.1, 95% CI = [21.3, 75.0], t(103) = 3.56, p = .001, BF = 36.6, gav = 0.196. This difference was again reliably

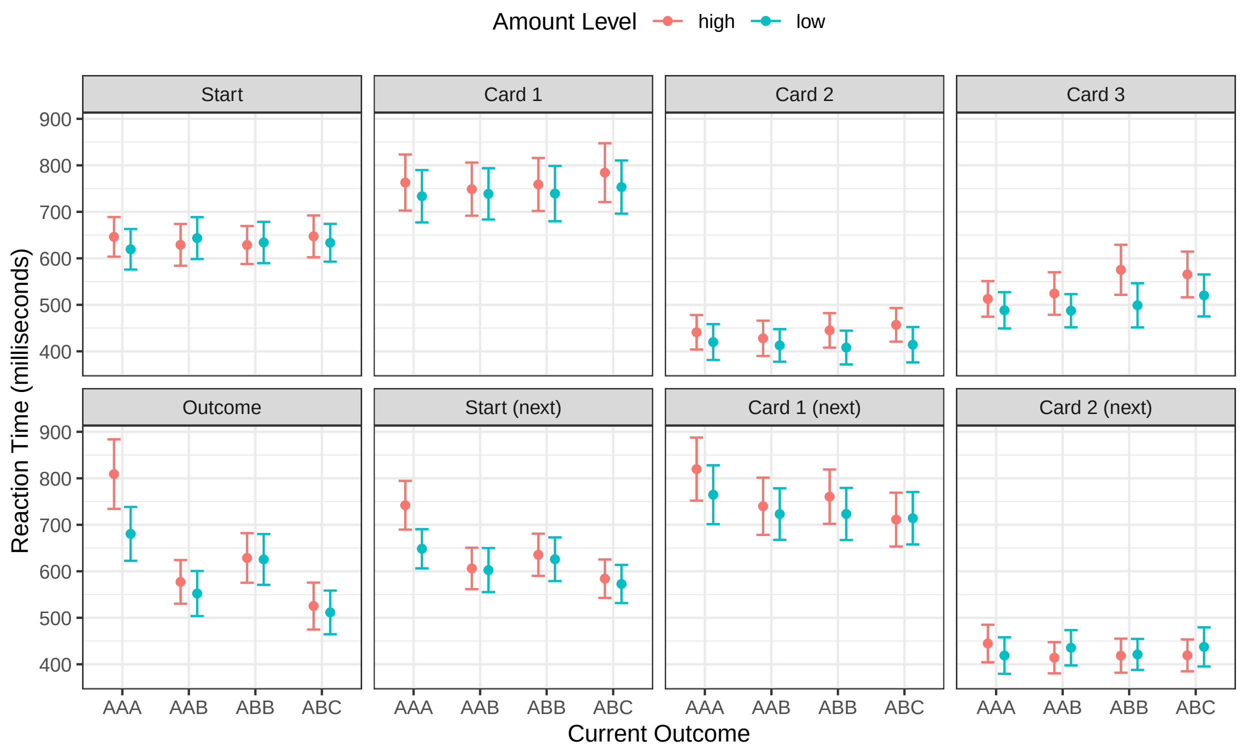

Figure S2

Reaction times of all eight responses within an 'episode' in Experiment 2. Error bars stand for 95% within-subjects confidence intervals.

larger than that in Experiment 1 of Chen et al. (2020), diff = 106.7, 95% CI = [68.2, 145.2], $t(140.6) = 5.48$, $p < .001$, BF = 81726, gav = 0.761.

For the new comparison between a win and a partial win with the same presented win amount, participants started a new round more slowly after $\text{AAA}_{2\text{-}10}$ ($M = 648.5$, $SD = 258$) compared to $\text{AAB/ABB}_{20\text{-}10}$ ($M = 620.9$, $SD = 257.9$), diff = 27.6, 95% CI = [3.5, 51.9], t(103) = 2.26, $p = 0.026$, BF = 1.23, gav = 0.107. However, the effect size was smaller than that observed on confirm RTs, and the Bayes factor was inconclusive, which might be due to that the start response was the second response following an outcome. Overall, the analyses on start RTs thus yielded the same pattern of results.

Table S2

Linear regression and pairwise comparisons on start RTs in Experiment 2.

Linear regression							
**Predictor**	**Estimate**	**SE**	**lowerCI**	**upperCI**	**t**	**p**	**BF**
Amount	29.55	7.69	14.46	44.64	3.84	< 0.001	180
Partial Win vs. Win	-77.71	9.42	-96.19	-59.23	-8.25	< 0.001	1.03×10^{13}
Partial Win vs. Loss	39.12	9.42	20.64	57.60	4.15	< 0.001	566
Amount * (Partial Win vs. Win)	-87.06	18.83	-124.02	-50.10	-4.62	< 0.001	4.52×10^{3}
Amount * (Partial Win vs. Loss)	-4.85	18.83	-41.81	32.11	-0.26	0.797	0.151
Pairwise comparisons							
**Comparison**	**diff**	**lowerCI**	**upperCI**	**t**	**p**	**BF**	**gav**
Win: High vs. Low	93.6	59.7	127.5	5.48	< 0.001	4.18×10^{4}	0.308
Partial Win: High vs. Low	6.6	-14.3	27.5	0.62	0.574	0.131	0.026
Loss: High vs. Low	11.4	-9.8	32.6	1.07	0.574	0.189	0.050

Note. lowerCI = lower limit of 95% confidence interval; upperCI = upper limit of 95% confidence interval; BF = Bayes factor; gav = Hedges's average g. P values for the pairwise comparisons were corrected for multiple comparisons with the Holm-Bonferroni method.

Experiment 3

Testing log

Data collection for Experiment 3 took place between 14-16 Dec 2021. 264 participants signed up and finished the experiment. 3 participants were timed out (no data recorded). 20 participants initially signed up but returned (no data recorded).

BFs during sequential sampling

Figure S3 showed the evolution of the Bayes factors during sequential sampling.

Memory of the payoff information

At the end of Experiment 3, participants were presented with the three types of games one by one, and asked to type in the wager and the presented 'win' amount for each outcome. To do the scratch card task, they did not need to remember this payoff

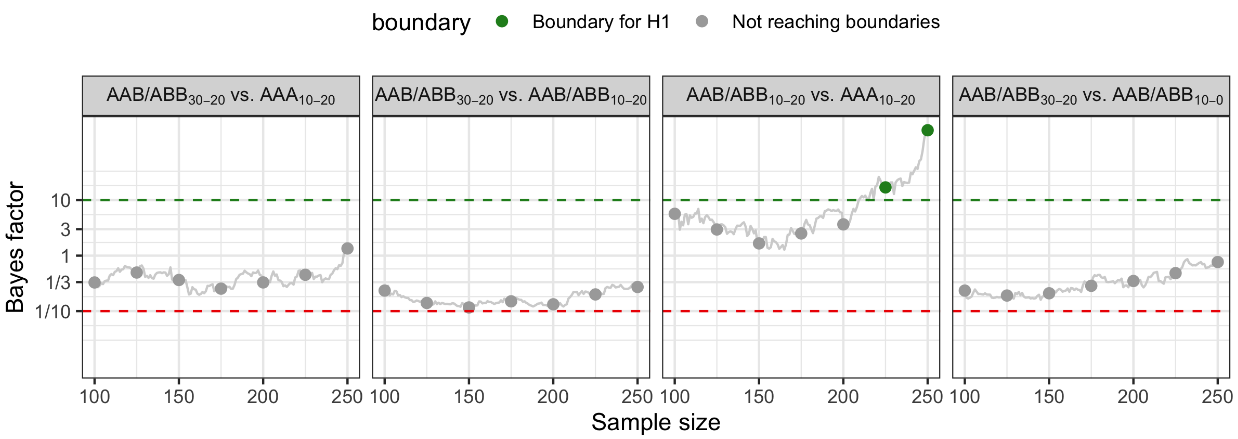

Figure S3

Bayes factors from the four planned comparisons during sequential testing in Experiment 3.

information, as the different 'win' amounts for the different outcomes were always presented on the top of the screen. We nevertheless explored the answers provided by participants, and examined whether these answers influenced the main comparisons of interest in a set of exploratory analyses.

Participants answered 12 questions in total (4 questions for each game). The memory data from one participant was missing, thus the sample contained 249 participants. The overall memory accuracy was high, M = 83.2%, SD = 19.8%. 1.74% of the answers were numbers that had never occurred in the task (e.g., one participant indicated winning 70 pence in a Type 1 game, while there was no 70-pence chip) and were therefore excluded. For the remaining answers, the proportion of each answer occurring for each question was computed and plotted (Figure S4).

Descriptively, the memory accuracy for the 'win' amount for AAB/ABB and AAA outcomes in Type 3 games was lower than those in Type 1 and Type 2 games. Note that AAB/ABB in Type 1 and 2 games were worth 20 pence, but 0 pence in Type 3 games. AAA in Type 1 and 2 games were worth 60 pence, but 20 pence in Type 3 games. The mismatch between Type 3 games, on the one hand, and Type 1 and Type 2 games, on the other hand, may explain why memory for AAB/ABB and AAA amount was reduced in

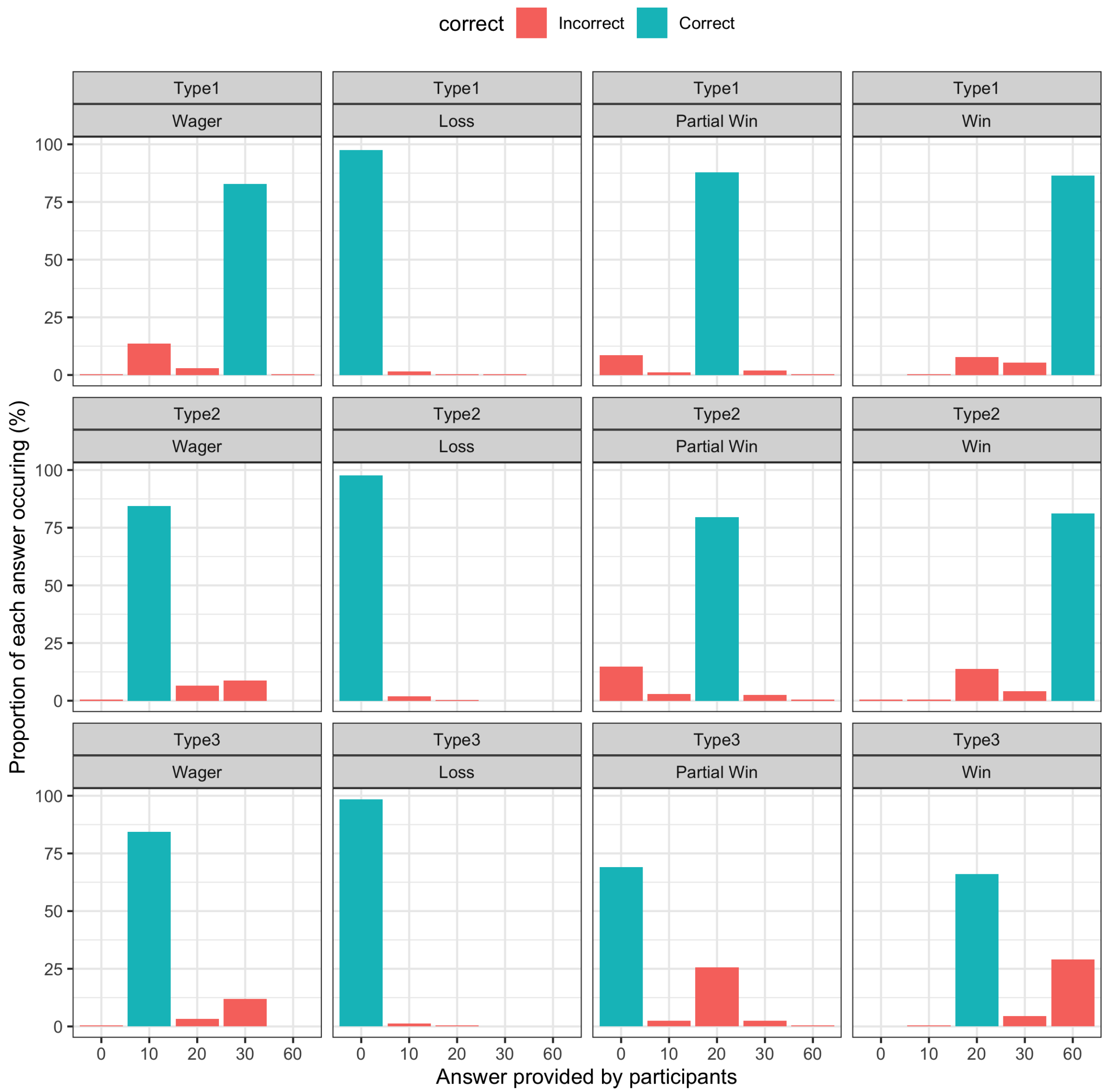

Figure S4

Proportion of answers provided by participants in the memory test in Experiment 3. The green bar stands for the correct answer for each question. Loss = ABC, Partial Win = AAB/ABB, Win = AAA.

Type 3 games. Note that for the loss amount, memory was highly accurate for all three types of games, as a loss (i.e., ABC) was always associated with 0 pence.

Next we examined whether memory would influence the four primary comparisons

reported in the main text. First, we selected participants who answered all 12 questions correctly. One hundred and seven participants met this criterion. Using this subset of participants, we repeated the four comparisons (Table S3). The results were descriptively all in line with the ones reported in the main text. While Comparison (3) between AAB/ABB$_{10\text{-}20}$ and AAA$_{10\text{-}20}$ was still statistically significant, the BF was no longer conclusive. For Comparison (4) between AAB/ABB$_{30\text{-}20}$ and AAB/ABB$_{10\text{-}0}$, the BF now provided moderate support for the alternative hypothesis, in the predicted direction.

Table S3

Pairwise comparisons on confirm RTs in Experiment 3 (N = 107 participants who answered all 12 questions correctly).

**Comparison**	**diff**	**lowerCI**	**upperCI**	**t**	**p**	**BF**	**gav**
AAB/ABB$_{30\text{-}20}$ vs. AAA$_{10\text{-}20}$	-29.2	-72.6	14.3	-1.33	0.344	0.253	0.076
AAB/ABB$_{30\text{-}20}$ vs. AAB/ABB$_{10\text{-}20}$	23.5	-10.3	57.2	1.38	0.344	0.268	0.062
AAB/ABB$_{10\text{-}20}$ vs. AAA$_{10\text{-}20}$	-52.6	-95.1	-10.2	-2.46	0.048	1.880	0.136
AAB/ABB$_{30\text{-}20}$ vs. AAB/ABB$_{10\text{-}0}$	48.2	14.9	81.4	2.87	0.020	5.171	0.133

Only including participants who answered all 12 questions correctly seemed to be a rather stringent requirement. Indeed, fewer than half of the 250 participants met this inclusion criterion. We thus repeated the analyses above, but this time using a more lenient criterion. That is, for each comparison, we included data from participants who correctly remembered the wagers and 'win' amounts for the current comparison (regardless of whether they remembered the payoff for other outcomes correctly or not). The sample sizes involved in each comparison thus differed. Again, the results were descriptively in line with the ones reported in the main text, and only Comparisons (3) and (4) were statistically reliable (Table S4).

Table S4

Pairwise comparisons on confirm RTs in Experiment 3 (participants who remembered the payoffs correctly for each comparison).

Comparison	N	diff	lowerCI	upperCI	t	p	BF	gav
$AAB/ABB_{30\text{-}20}$ vs. $AAA_{10\text{-}20}$	147	-29.9	-71.8	12.0	-1.41	0.322	0.242	0.075
$AAB/ABB_{30\text{-}20}$ vs. $AAB/ABB_{10\text{-}20}$	139	17.8	-13.3	48.9	1.13	0.322	0.176	0.043
$AAB/ABB_{10\text{-}20}$ vs. $AAA_{10\text{-}20}$	122	-55.0	-96.1	-13.9	-2.65	0.028	2.821	0.138
$AAB/ABB_{30\text{-}20}$ vs. $AAB/ABB_{10\text{-}0}$	148	39.3	10.9	67.8	2.73	0.028	3.256	0.100

Wager amount in appraisals

Table S5

Exploratory pairwise comparisons on the effect of wager amount on confirm RTs and start RTs in Experiment 3.

Exploratory analyses on confirm RTs							
**Comparisons**	**diff**	**lowerCI**	**upperCI**	**t**	**p**	**BF**	**gav**
(1) $AAA_{30\text{-}60}$ vs. $AAA_{10\text{-}60}$	-33.1	-65.2	-1.1	-2.04	0.043	0.542	0.073
(2) $ABC_{30\text{-}0}$ vs. $ABC_{10\text{-}0}$ (Type 2)	-6.5	-34.7	21.7	-0.46	0.649	0.078	0.018
(3) $ABC_{30\text{-}0}$ vs. $ABC_{10\text{-}0}$ (Type 3)	0.7	-26.7	28.0	0.05	0.962	0.071	0.002
Exploratory analyses on start RTs							
**Comparisons**	**diff**	**lowerCI**	**upperCI**	**t**	**p**	**BF**	**gav**
(1) $AAA_{30\text{-}60}$ vs. $AAA_{10\text{-}60}$	-35.0	-70.2	0.2	-1.96	0.051	0.474	0.076
(2) $ABC_{30\text{-}0}$ vs. $ABC_{10\text{-}0}$ (Type 2)	-50.4	-76.2	-24.6	-3.85	< 0.001	87.2	0.135
(3) $ABC_{30\text{-}0}$ vs. $ABC_{10\text{-}0}$ (Type 3)	-81.0	-108.8	-53.2	-5.74	< 0.001	313914	0.214

Note. lowerCI = lower limit of 95% confidence interval; upperCI = upper limit of 95% confidence interval; BF = Bayes factor; gav = Hedges's average g. P values were **not** corrected for multiple comparisons.

To further explore whether participants incorporated the wager amount into their appraisals of different outcomes, we selected pairs where both the card configuration and

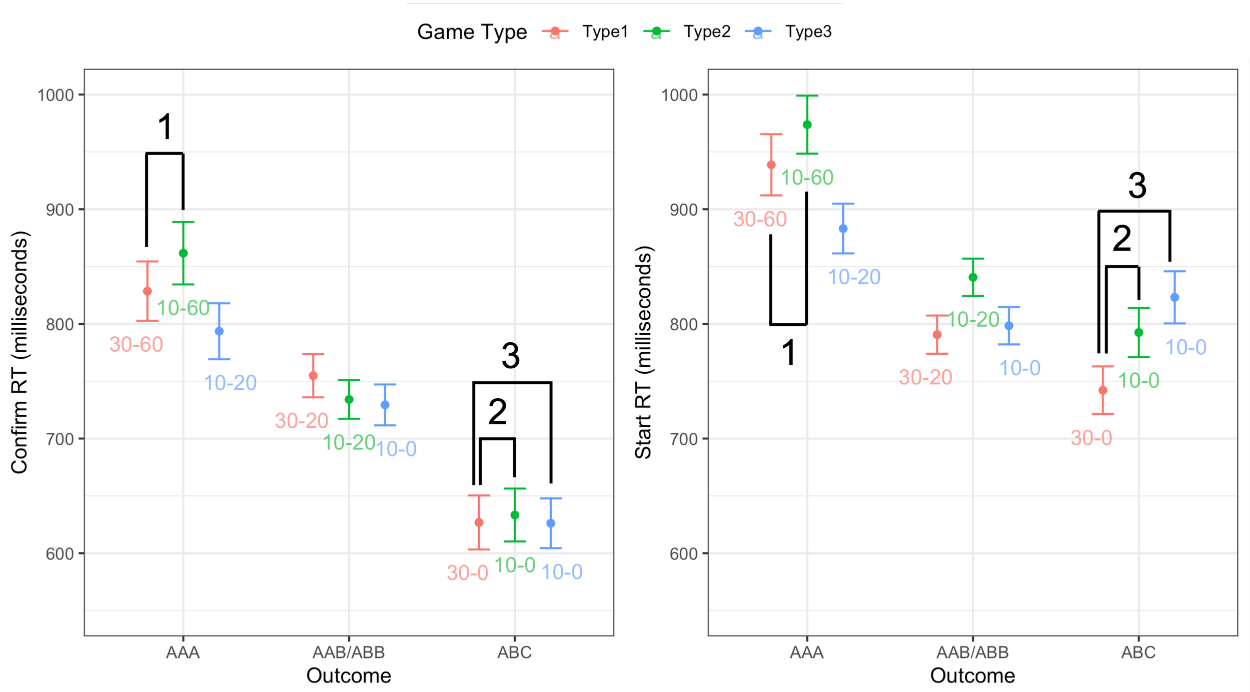

Figure S5

Confirm RT (left) and Start RT (right) as a function of outcome and game type in Experiment 3. Error bars stand for 95% within-subjects confidence intervals. The numbers in red, green and blue stand for the wager and the presented win amount in each game (wager-win). The numbers 1-3 in black show the two cells involved in each of the three comparisons (see Table S5 for the corresponding results).

the presented 'win' amount was matched, with only the wager amount differed. Three new pairs were compared (see Figure S5 and Table S5). For Comparison (1), participants responded to $AAA_{30\text{-}60}$ more quickly than $AAA_{10\text{-}60}$, for both confirm RTs and start RTs. However, the p values were close to .05, and the BFs were inconclusive. There was thus some evidence that participants incorporated the wager amount when appraising wins.

Response vigor after losses showed a different pattern. When confirming losses, wager amount had no effect. However, when starting a new trial, wager amount had reliable effects (Comparisons (2) and (3)). Participants started a new trial more quickly after $ABC_{30\text{-}0}$ than $ABC_{10\text{-}0}$. This result is in line with that observed for AAB/ABB in the

manuscript (Comparison (3), between AAB/ABB$_{30\text{-}20}$ and AAB/ABB$_{10\text{-}20}$). When the wager was made salient as in Experiment 3, participants thus could incorporate the wager amount into their appraisals of outcomes, but this might take some time (at least for AAB/ABB and ABC outcomes).

Presented 'win' amount in appraisals

In another set of exploratory analyses, we evaluated the effect of presented 'win' amount on response vigor, with both the wager amount and the card configurations matched. We identified and compared two pairs (see Figure S6).

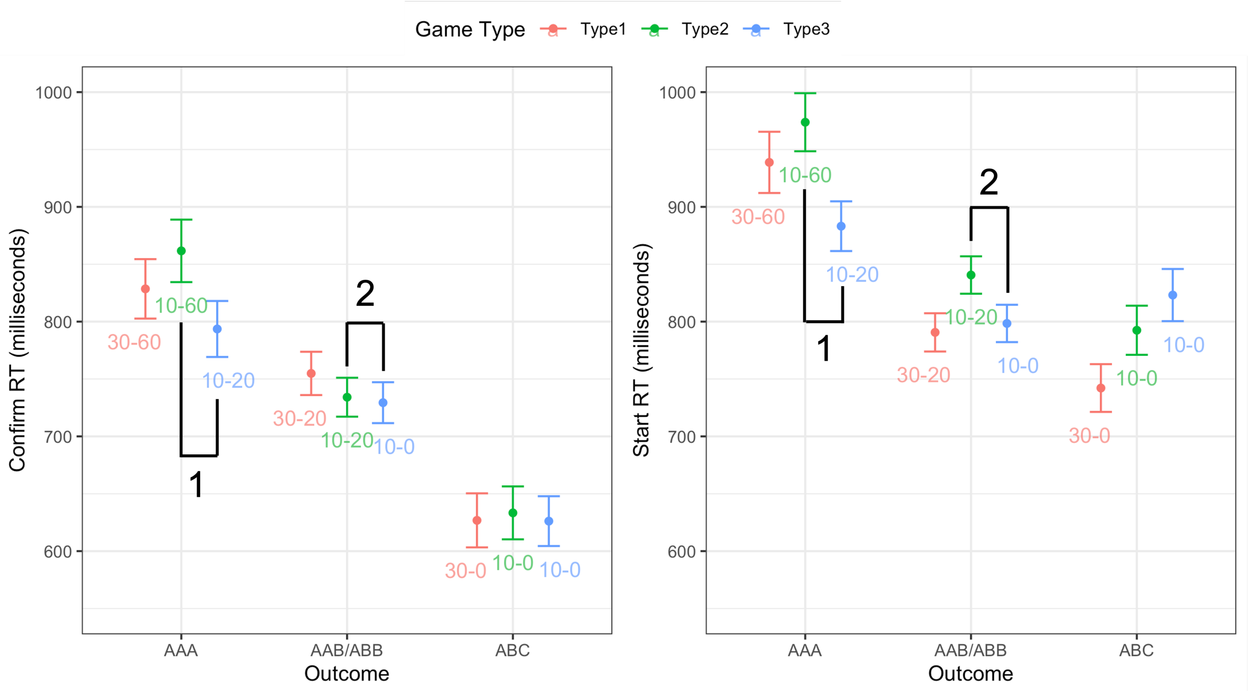

Figure S6

Confirm RT (left) and Start RT (right) as a function of outcome and game type in Experiment 3. Error bars stand for 95% within-subjects confidence intervals. The numbers in red, green and blue stand for the wager and the presented win amount in each game (wager-win). The numbers 1-2 in black show the two cells involved in each of the three comparisons (see Table S6 for the corresponding results).

Table S6

Exploratory pairwise comparisons on the effect of presented 'win' amount on confirm RTs and start RTs in Experiment 3.

Exploratory analyses on confirm RTs							
**Comparisons**	**diff**	**lowerCI**	**upperCI**	**t**	**p**	**BF**	**gav**
(1) $AAA_{10\text{-}60}$ vs. $AAA_{10\text{-}20}$	68.1	37.5	98.6	4.39	< 0.001	682.7	0.155
(2) $AAB/ABB_{10\text{-}20}$ vs. $AAB/ABB_{10\text{-}0}$	4.8	-18.7	28.2	0.40	0.690	0.077	0.013
Exploratory analyses on start RTs							
**Comparisons**	**diff**	**lowerCI**	**upperCI**	**t**	**p**	**BF**	**gav**
(1) $AAA_{10\text{-}60}$ vs. $AAA_{10\text{-}20}$	90.6	58.8	122.4	5.62	< 0.001	1.67×10^5	0.207
(2) $AAB/ABB_{10\text{-}20}$ vs. $AAB/ABB_{10\text{-}0}$	42.2	20.2	64.2	3.78	< 0.001	68.5	0.108

Note. lowerCI = lower limit of 95% confidence interval; upperCI = upper limit of 95% confidence interval; BF = Bayes factor; gav = Hedges's average *g*. P values were **not** corrected for multiple comparisons.

The results on confirm RTs were broadly in line with those observed in Experiments 1 and 2. While confirm RTs after AAA were modulated by the win amount, confirm RTs after AAB/ABB were not. For start RTs, the results showed a different pattern. Start RTs after both AAA and AAB/ABB were modulated by the presented 'win' amount. In line with the exploratory analyses presented in the previous section, for the best outcome (i.e., AAA), participants incorporated the presented 'win' amount into the appraisal, while for other outcomes (i.e., AAB/ABB) it might take some time.
